# Small-Quantity Lipid-Based Nutrient Supplements, Regardless of Their Zinc Content, Increase Growth and Reduce the Prevalence of Stunting and Wasting in Young Burkinabe Children: A Cluster-Randomized Trial

**DOI:** 10.1371/journal.pone.0122242

**Published:** 2015-03-27

**Authors:** Sonja Y. Hess, Souheila Abbeddou, Elizabeth Yakes Jimenez, Jérôme W. Somé, Stephen A. Vosti, Zinéwendé P. Ouédraogo, Rosemonde M. Guissou, Jean-Bosco Ouédraogo, Kenneth H. Brown

**Affiliations:** 1 Program in International and Community Nutrition, Department of Nutrition, University of California Davis, Davis, CA, United States of America; 2 Nutrition Program, Department of Individual, Family and Community Education, University of New Mexico, Albuquerque, NM, United States of America; 3 Institut de Recherche en Sciences de la Santé, Bobo-Dioulasso, Burkina Faso; 4 Agricultural and Resource Economics, University of California Davis, Davis, CA, United States of America; Burnet Institute, AUSTRALIA

## Abstract

Small-quantity lipid-based nutrient supplements (SQ-LNS) are promising home fortification products, but the optimal zinc level needed to improve growth and reduce morbidity is uncertain. We aimed to assess the impact of providing SQ-LNS with varied amounts of zinc, along with illness treatment, on zinc-related outcomes compared with standard care. In a placebo-controlled, cluster-randomized trial, 34 communities were stratified to intervention (IC) or non-intervention cohorts (NIC). 2435 eligible IC children were randomly assigned to one of four groups:1) SQ-LNS without zinc, placebo tablet; 2) SQ-LNS containing 5mg zinc, placebo tablet; 3) SQ-LNS containing 10mg zinc, placebo tablet; or 4) SQ-LNS without zinc and 5mg zinc tablet from 9–18 months of age. During weekly morbidity surveillance, oral rehydration salts were provided for reported diarrhea and antimalarial therapy for confirmed malaria. Children in NIC (n = 785) did not receive SQ-LNS, tablets, illness surveillance or treatment. At 9 and 18 months, length, weight and hemoglobin were measured in all children. Reported adherence was 97±6% for SQ-LNS and tablets. Mean baseline hemoglobin was 89±15g/L. At 18 months, change in hemoglobin was greater in IC than NIC (+8 vs -1g/L, p<0.0001), but 79.1% of IC were still anemic (vs. 91.1% in NIC). Final plasma zinc concentration did not differ by group. During the 9-month observation period, the incidence of diarrhea was 1.10±1.03 and of malaria 0.54±0.50 episodes per 100 child-days, and did not differ by group. Length at 18 months was significantly greater in IC compared to NIC (77.7±3.0 vs. 76.9±3.4cm; p<0.001) and stunting prevalence was significantly lower in IC (29.3%) than NIC (39.3%; p<0.0001), but did not differ by intervention group within IC. Wasting prevalence was also significantly lower in IC (8.7%) than in NIC (13.5%; p = 0.0003). Providing SQ-LNS daily with or without zinc, along with malaria and diarrhea treatment, significantly increased growth and reduced stunting, wasting and anemia prevalence in young children.

**Trial Registration:**

ClinicalTrials.gov NCT00944281

## Introduction

Growth stunting in early life continues to be a critical public health concern in low-income countries [1,2]. Diarrhea and pneumonia are leading causes of morbidity and mortality among young children in low income countries and the case fatality for these illnesses is increased in undernourished children [3,4]. Although the global malaria burden has decreased in the past decade, it remains very high in sub-Saharan Africa [5]. Supplemental zinc increases linear growth and weight gain and reduces the incidence of diarrhea and pneumonia among children under 5 years of age [6–10]. Small quantity lipid-based nutrient supplements (SQ-LNS) have been designed to prevent undernutrition and promote health and development [11]. SQ-LNS provide ~20 g or ~110–120 kcal per day along with additional protein, essential fatty acids and 22 micronutrients, including zinc. Trials investigating the potential of SQ-LNS and medium-quantity LNS (MQ-LNS; at ~50 g or ~250–280 kcal per day) to prevent malnutrition and promote growth have yielded mixed results [12–17]. Only a few of these studies reported morbidity outcomes, and none detected any benefit of SQ-LNS [14,15]. Thus, the beneficial impact of zinc on growth and morbidity is less clear when provided mixed with food, such as point-of-use fortification of complementary foods with SQ-LNS or multiple micronutrient powders (MNP), possibly because of insufficient zinc content.

While there is strong evidence that providing 3–20 mg zinc supplements daily as syrup or tablets consistently results in significantly increased plasma zinc concentrations (pZn) in the zinc supplemented group compared to the placebo control group [18], findings from home fortification trials are inconsistent. Most, but not all, studies providing 5 mg zinc in a daily dose of SQ-LNS or MNP did not find increased pZn at the end of the study [19–21]. One study in young Iranian children who received 5 mg of zinc in MNP and one study in young Cambodian children who received 10 mg of zinc in MNP found a significantly increased pZn at the end of the study compared to a placebo control group and non-intervention group, respectively [22,23]. Possible explanations for this lack of response in biochemical and functional outcomes are: 1) the dose of zinc was inadequate because less zinc is absorbed from zinc-containing preparations that are provided with meals than from water-soluble zinc supplements provided between meals, 2) the study populations were not zinc deficient, so they did not respond functionally to additional zinc, and/or 3) zinc consumed with meals was taken up preferentially by non-metabolically active zinc pools.

The present study had two primary objectives: 1) To compare zinc-related functional responses among young Burkinabe children who received SQ-LNS containing 0, 5 or 10 mg of zinc and placebo tablet or SQ-LNS without added zinc, plus 5 mg zinc tablets along with treatment for diarrhea and malaria episodes; and 2) to compare the impact of the intervention package among children in intervention vs. non-intervention communities. Cluster-randomization at the level of the family compound (i.e. concession) in the intervention communities was used to prevent cross-contamination between intervention groups through food sharing. Major outcomes included physical growth, morbidity from common infections (specifically, diarrhea and malaria), and change in pZn.

## Materials and Methods

The protocol for this trial and supporting CONSORT checklist are available as supporting information; see S1 CONSORT Checklist and S1 Protocol.

### Study Design and Participants

The study was designed as a partially masked, placebo-controlled, cluster-randomized intervention trial. The trial was conducted from April 2010 to July 2012 in rural communities of the Dandé Health District in southwestern Burkina Faso. Ethical approval for the study protocol and the consent procedure was provided by the Institutional Review Boards of the Centre Muraz in Bobo-Dioulasso (Burkina Faso) and the University of California Davis (USA). The study was registered as a clinical trial with the U.S. National Institutes of Health (NCT00944281;).

Nine-month old infants were identified by periodic censuses in the study area. Written, informed consent was obtained from one of the child’s primary caregivers (mother, father or legal guardian). In case the caregiver was illiterate, an impartial witness was present during the consent process, who confirmed that the information in the consent document was accurately explained to the participant, and that consent was freely given. Children were considered eligible if they were 8.8 to 9.9 months of age, resided permanently in the area, planned to be available during the study period and had written parental consent. Exclusion criteria were: hemoglobin (Hb) <50 g/L, weight-for-length <70% of the median of the National Center for Health Statistics/World Health Organization (NCHS/WHO) growth reference [24] presence of bipedal edema, other severe illness warranting hospital referral, congenital abnormalities potentially interfering with growth, chronic medical conditions requiring frequent medical attention, known HIV infection of infant or mother, history of allergy towards peanuts, history of anaphylaxis or serious allergic reaction to any substance requiring emergency medical care, and concurrent participation in any other clinical trial. Once enrolled, children were withdrawn from study participation when absent from the study for more than 3 weeks, although data collected until that time were retained.

### Sample Size

The sample size estimates were based on the number of children needed in each group to detect (with a significance level of P<0.05 and power >0.80) effect sizes that are consistent with the magnitude of effects observed in previous zinc supplementation trials [6,25]. In particular, the sample sizes were based on an effect size of >0.22 for diarrhea incidence, malaria incidence, and physical growth; and 0.6 for change in pZn. The target sample size per intervention group for incidence of diarrhea and malaria and growth was n = 583 and for biochemical indicators of nutritional status n = 85. This included an assumed attrition rate of 15% for morbidity and growth outcomes and 20% for the biochemical outcomes. The estimated sample size for the NIC was increased for an assumed design effect of 1.5 due to the cluster sampling design.

### Randomization and Masking

This trial included two levels of randomization: 1) the community and 2) the concession. Thirty-four communities that were accessible year-round were chosen. A statistician at the University of California Davis stratified individual communities by selected indicators (population size; proximity to road and Bobo-Dioulasso; and health clinic affiliation) and computer-generated an assignment within strata to participate in the intervention cohort (IC; 25 communities) or in the non-intervention cohort (NIC; 9 communities). The same statistician, who was blinded to the intervention, generated a random allocation sequence at the level of the concession for the enrollment of eligible infants in the IC. Eligible children meeting the inclusion criteria were allocated to one of four interventions from 9 to 18 months of age: 1) SQ-LNS without zinc, and placebo tablet (LNS-Zn0), 2) SQ-LNS with 5 mg zinc, and placebo tablet (LNS-Zn5), 3) SQ-LNS with 10 mg zinc, and placebo tablet (LNS-Zn10), or 4) SQ-LNS without zinc, and 5 mg zinc tablet (LNS-TabZn5). Children in the intervention groups received free treatment for diarrhea, malaria and fever, as described in more detail below. Children in the NIC did not receive SQ-LNS or tablets from 9 to 18 months of age, nor any illness treatment, but received SQ-LNS from 18 to 27 months after the data collection was finished. The trial was partially masked, as all participants, field staff and researchers remained blinded to the four intervention groups until data analyses were completed, but were aware which communities were assigned to IC and NIC.

The LNS products were developed for the iLiNS project [11] by Nutriset SAS (Malaunay, France) and tested for acceptability [26]. A weekly ration of LNS was initially delivered to participating children in plastic cups containing 140 g (sufficient for one week) and later in seven sachets containing 20 g each. The child’s caregiver was advised to feed the day’s allotment (20 g = 2 spoonsful) mixed in food during two separate meal times. The LNS for each treatment group were identical, except for their zinc content. Besides zinc, a daily ration of LNS provided 118 kcal and 6 mg iron along with 20 other micronutrients [11]. Table 1 shows the nutrient content of a daily ration of SQ-LNS compared with the Recommended Nutrient Intakes by WHO and the Food and Agriculture Organization of the United Nations (FAO) [27,28]. Zinc tablets were provided as water-dispersible tablets produced by Nutriset SAS (Malaunay, France) containing 5 mg zinc or an identical placebo. The caregivers were advised to provide the tablet once daily, dissolved in water or breast milk, but not with other foods. SQ-LNS and packages of tablets were labeled with one of eight color codes (two colors per intervention group). Caregivers were given brief feeding advice at enrollment, which included the above-described instructions for SQ-LNS and tablets and recommendations to continue breastfeeding and to provide a variety of nutritious foods.

**Table 1:** Nutrients provided in 20 g SQ-LNS for infants and young children, in comparison to World Health Organization/Food and Agriculture Organization (WHO/FAO) Recommended Nutrient Intakes (RNI) [27].

Nutrient	Unit	SQ-LNS	WHO/FAO RNI	
			7–12 months	1–3 yrs
Energy	kcal	118	-	-
Protein	g	2.6	10.5	11.9
Fat	g	9.6	40–60% energy^1^	
Linoleic acid	G	4.46	3.0–4.5% energy	
α-Linolenic acid	G	0.58	0.4–0.6% energy	
Vitamin A	Μg	400	400	400
Thiamin (B1)	Mg	0.3	0.3	0.5
Riboflavin (B2)	Mg	0.4	0.4	0.5
Niacin (B3)	Mg	4	4	6
Pantothenic acid (B5)	Mg	1.8	1.8	2
Vitamin B6	Mg	0.3	0.3	0.5
Vitamin B12	Μg	0.5	0.7	0.9
Folic acid	Μg	80	80	150
Vitamin C	Mg	30	30	30
Vitamin D	Μg	5	5	5
Vitamin E	Mg	6	2.7	5
Vitamin K	Μg	30	10	15
Calcium	Mg	280	400	500
Copper	Mg	0.34	-	-
Iodine	Μg	90	90	90
Iron	Mg	6	18.6^2^	11.6^2^
Magnesium	Mg	40	54	60
Manganese	Mg	1.2	-	-
Phosphorus	Mg	190	-	-
Potassium	Mg	200	-	-
Selenium	μg	20	10	17
Zinc	mg	0, 5 or 10^3^	8.4^4^	8.3^4^

^1^ Acceptable macronutrient distribution range recommended to reduce gradually from 40–60% at 6 months to 35% at 24 months and to 25–35% for 2 years and older [28].

^2^ RNI for an assumed bioavailability of 5%.

^3^ Zinc content depending on intervention group.

^4^ RNI for low bioavailability.

### Procedures

At the time of enrollment, children’s length, weight, mid-upper arm circumference (MUAC) and head circumference (HC) were measured in IC and NIC. Maternal height and weight, and information on the child’s dietary practices and family socio-economic status (SES) were also obtained for all children. The age of the participants was confirmed from health cards. A capillary sample was collected for onsite analyses of Hb concentration (Hemocue 201+, HemoCue AB, Ängelholm, Sweden) and a rapid diagnostic test (RDT) for malaria parasites, based on histidine-rich protein II (SD BIOLINE Malaria Ag P.F/Pan, Standard Diagnostics, INC., Kyonggi-do, Korea). Capillary blood (300 μL) was collected in microcuvettes containing lithium heparin (CB 300 LH, Sarstedt AG & Co, Nümbrecht, Germany) for assessment of zinc protoporphyrin (ZPP) concentration. In case of illness, all children screened received free medical treatment(s): in case of diarrhea oral rehydration salts (ORS) packets were provided, children with fever received paracetamol and children with a positive RDT received anti-malarial treatment (artesunate-amodiaquine combination therapy and paracetamol). Children with Hb <80 g/L received anthelmintic treatment (mebendazole 200 mg/day for 3 days) and iron supplements (2–6 mg iron/kg body weight for 30 days). Children with weight-for-length <70% of the median of the NCHS/WHO growth reference [24] or with malaria or diarrhea with complications or any other illness requiring medical follow up were referred to the health center. Enrollment continued until the target sample size was met. In the case of twins, both were enrolled in the study, but only one was randomly selected for inclusion in the data analysis.

Children in the IC were visited weekly for delivery of intervention products and morbidity surveillance using standardized data collection tools. During each visit, reported LNS and tablet consumption was recorded, and empty and unused packages were collected for assessment of adherence. At each visit, the field worker evaluated the child for the presence of clinical danger signs, and evidence of uncomplicated diarrhea, fever, or malaria, which were treated in the community, as described above. In case of reported fever on the day of or the day before the surveillance visit, the field worker performed an RDT and measured aural body temperature. Children with danger signs, and diarrhea, fever and malaria with complications and any other cases of severe illness were referred to the health center for evaluation and treatment. Surveillance field workers participated in regular training, and refresher sessions and were overseen by field supervisors and two study physicians. A randomly selected sub-sample of children participated in 12-hr home observations at 11 and 16 months of age, as described in more detail elsewhere [29].

The anthropometric measurements described above were repeated at 12 and 15 months in IC only. From the age of 9 to 18 months, children in NIC were not supplemented nor visited by study personnel; they relied on standard care provided by the family and health system. At 18 months, all children (IC and NIC) were invited for a final follow up examination, where anthropometric assessments were repeated, and Hb concentration was assessed. Children’s and mothers’ weights were measured to 50 g precision (Seca Model 383 and Seca Model 874) and children’s length (Seca Model 417), MUAC (26 cm Tri-Colored Single-Slotted Insertion Tape), HC (ShorrTape Measuring Tape), and mothers’ height (Seca Model 217) were measured to 0.1 cm precision. Four teams of two anthropometrists each completed measurements in both IC and NIC and were systematically restandardized. During 12 standardization sessions, the mean technical error of measurement (TEM) for length was 0.50 cm and ranged from 0.09 to 0.74 cm (N = 120) considering both intra- and inter-observer variability [30].

At enrollment, a subset of children in IC and NIC were randomly assigned to the “biochemistry sub-group” for a venous blood draw. To avoid intra-concession correlation in this sub-group, only one child was randomly selected per family compound. Final venous blood samples were obtained from the same sub-set of children at 18 months of age. On both occasions, children were free of symptoms of diarrhea and fever during the previous 48 hr, and 5 mL of blood was drawn 1–2 hr after the last breastfeeding episode from an antecubital or dorsal metacarpal vein. Blood was collected in evacuated, trace element-free polyethylene tubes containing lithium heparin (Sarstedt AG & Co, Nümbrecht, Germany), stored on ice and transported to the field laboratory, where ZPP was assessed in unwashed capillary whole blood by hematofluorometer (206D, AVIV Biomedical Inc., Lakewood, NJ, USA). Plasma was separated from heparinized venous blood by centrifuging at 2800 rpm for 10 min and stored at -20°C until shipped on dry ice to the respective laboratories for analysis. All blood collection and processing procedures conformed with recommendations by the International Zinc Nutrition Consultative Group (IZiNCG) [31]. pZn was determined by inductively-coupled plasma atomic emission spectrometry following overnight digestion in 70% nitric acid at the Children’s Hospital Oakland Research Institute [32,33]. C-reactive protein (CRP) and α-1-acid glycoprotein (AGP) were analyzed by ELISA by DBS-Tech in Willstaett, Germany [34].

### Outcomes

Length-for age (LAZ), weight-for-age (WAZ), and weight-for-length z-scores (WLZ) were calculated according to the World Health Organization 2006 growth standards [35]. Stunting, underweight and wasting were defined as <-2 standard deviation (SD) LAZ, WAZ and WLZ, respectively. Diarrhea was defined as the presence of ≥3 loose or liquid stools per 24 hr. Reported fever was defined as any fever reported by the caregiver. Confirmed fever was defined as aural temperature >37.5°C. Any episode of reported or confirmed fever associated with a positive RDT was defined as malaria. A child was considered at risk for malaria if the child did not receive any antimalarial treatment within the previous 21 days. New episodes of diarrhea and fever were considered when at least 48 hr had elapsed since a previous episode. To assess food security, the Household Food Insecurity Access Scale (HFIAS [36]) was adjusted for season and year. Using principal components analysis, an asset index was computed from information on baseline ownership of a set of assets, lighting source, drinking water supply, sanitation facilities, and flooring materials [37]. pZn and ZPP concentrations were adjusted for the presence of inflammation (elevated acute phase proteins categorically defined as CRP >5 mg/L, AGP >1 g/L, or both). Cut-offs used to define deficiency for each indicator were: pZn < 65 μg/dL; Hb < 110 g/L; ZPP >70 μmol/mol heme.

### Statistical Analyses

The primary outcomes were growth, incidence of diarrhea, incidence of malaria and pZn. All statistical analyses were completed with SAS System software for Windows release 9.2 (SAS Institute, Cary, North Carolina, USA). Data are presented as means ± SD, unless otherwise noted. Treatment groups remained masked until all statistical analyses were completed and main conclusions were drawn.

Descriptive statistics were used to examine all variables. Because the method of allocating communities to the two cohorts did not result in equal probabilities of being selected for intervention, selected variables were compared between cohorts using linear mixed models for continuous variables, mixed logistic regression models for binary variables, including random effects of village and concession, and Chi-square tests accounting for community for non-binary categorical variables. Covariates and potential effect modifiers were defined prior to analyses. Linear mixed model ANCOVA (SAS MIXED procedure) was used to compare anthropometric outcomes, and Hb, pZn, AGP and CRP concentrations between the two cohorts, among the four intervention groups and among the five groups (four intervention groups and NIC) with adjustment for baseline value, age, other relevant variables and random effects (village, concession, if appropriate). Differences in the prevalence of stunting, underweight, and wasting at the end of the study were compared with mixed model logistic regression (SAS GLIMMIX procedure) adjusting for respective baseline z-score and other co-variates. Potential modifying effects with an interaction with the treatment group and/or cohort were considered significant if P<0.1. The modifying effects of baseline LAZ on the change in LAZ was examined by estimating adjusted means at the 10^th^, 50^th^ and 90^th^ percentile of baseline LAZ.

Morbidity outcomes were compared by using mixed effects logistic regression (SAS GLIMMIX procedure), including random effects of concession, and accounting for overdispersion. Treatment of diarrhea included treatments provided both by project field workers and health centers. For treatment of malaria, only the treatment provided by field workers was considered. Differences in mortality rates between enrollment and end of the study were compared with logistic regression including random effects of village and concession.

## Results

**Fig 1:**
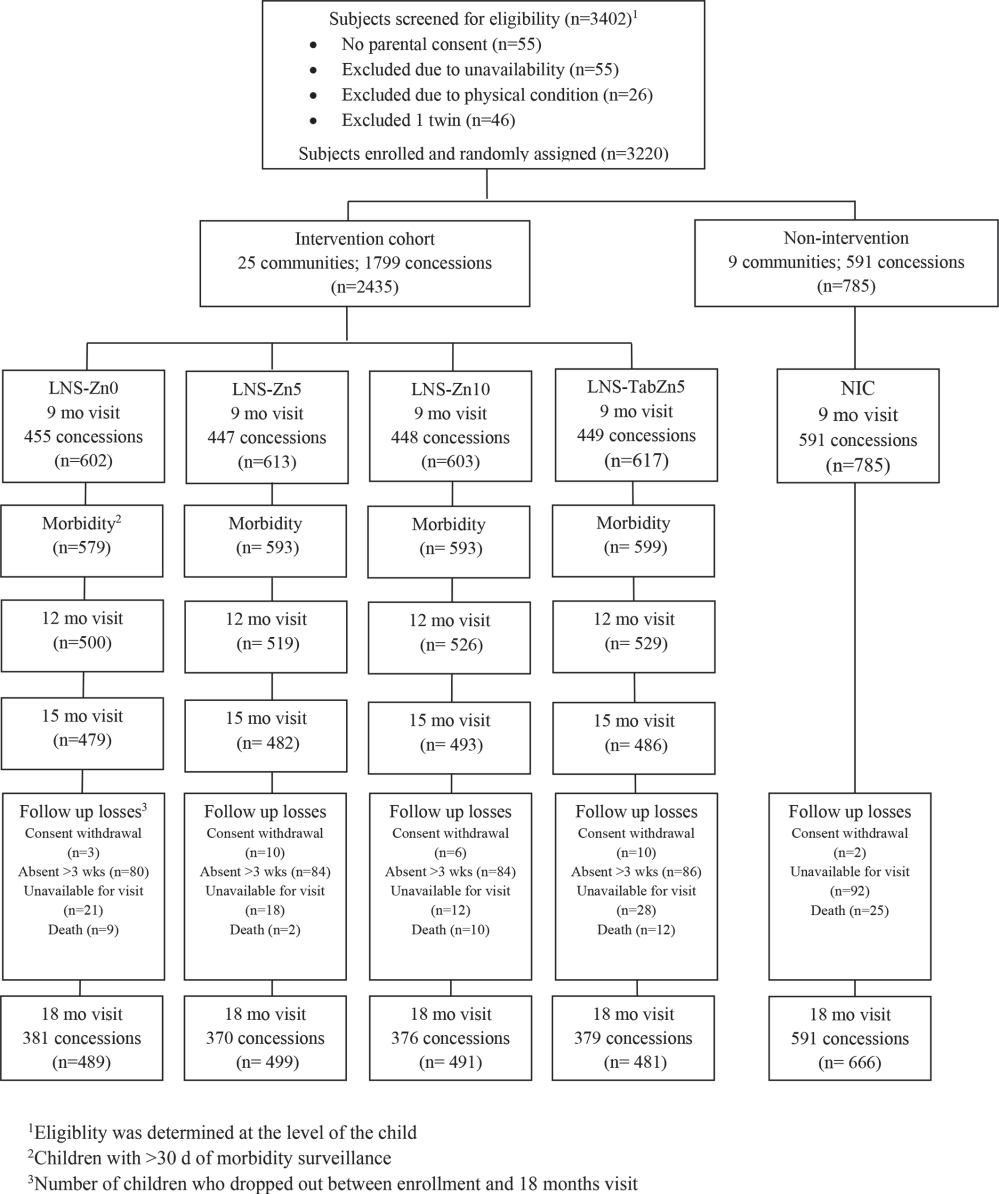
Flow diagram of clusters and participant progression through the iLiNS-ZINC trial.

Of 3402 children screened for eligibility, 3220 were enrolled in the study (**Fig. 1**). Of these, 2435 were randomly assigned to one of the 4 treatment groups in the IC and 785 to the NIC. Among the four intervention groups, 19.5% dropped out by the final visit at 18 months. In the NIC, 15.2% were lost to anthropometric follow up at 18 months. Caregivers of 97.1% of children in the intervention groups provided morbidity surveillance information for at least 30 days and 78% provided information for at least 35 weeks, with no difference in participation rates among the four intervention groups.

Potential biases associated with loss to follow up were assessed. There were no differences in child, maternal, or household characteristics or SES between children who completed the study and those who dropped out, except for maternal age. Mothers of children who completed the study were slightly older than those of non-completers (27.0 ± 6.7 vs. 26.2 ± 6.7; P = 0.004).

At enrollment, children had a mean age of 9.4 ± 0.35 months, and almost all children (99.9%) were still breastfed. Most baseline child and maternal characteristics did not differ among the groups (Table 2). However, WAZ and WLZ of children in LNS-Zn10 were significantly lower than in children in the LNS-TabZn5 group at baseline. There was also a significant difference in maternal education and marital status/rank. More women in NIC had no education (67.6%) compared to women in the 4 intervention groups (56.2–60.8%). Although other SES characteristics were not significantly different between groups, there was a tendency towards lower SES in the NIC.

**Table 2:** Child and maternal baseline characteristics by group.

	LNS-Zn0	LNS-Zn5	LNS-Zn10	LNS-TabZn5	NIC	P-value^1^
N	602	613	603	617	785	
**Child characteristics**						
Age (months) ^2^	9.4 ± 0.35	9.4 ± 0.35	9.4 ± 0.35	9.4 ± 0.36	9.4 ± 0.35	0.447
Male	305 (51)	299 (49)	320 (53)	304 (49)	392 (50)	0.600
Hemoglobin (g/L)	90 ± 15	90 ± 16	89 ± 15	90 ± 15	89 ± 16	0.747
Adjusted ZPP^3^ (μmol/mol heme)	168.1 (161.1, 175.5)	175.0 (168.0, 182.2)	172.2 (165.0, 179.7)	179.1 (171.6, 186.8)	168.8 (1622, 175.7)	0.293
Positive RDT^4^	369 (61)	358 (58)	368 (61)	351 (57)	532 (68)	0.244
Still breastfed	600 (100)	612 (99.8)	603 (100)	617 (100)	784 (99.9)	1.000
**Maternal characteristics**						
Maternal age (yrs)	26.6 ± 6.5	27.2 ± 6.7	26.9 ± 6.8	26.9 ± 7.1	26.8 + 6.5	0.669
Maternal height (cm)	162.3 ± 5.9	162.3 ± 5.7	161.7 ± 5.5	162.0 ± 5.6	162.5 ± 5.8	0.195
Maternal weight (kg)	55.1 ± 7.8	55.3 ± 7.4	54.8 + 7.8	55.3 ± 8.2	54.5 ± 7.5	0.797
Maternal BMI (kg/m^2^)	20.9 ± 2.5	21.0 ± 2.4	20.9 ± 2.6	21.0 ± 2.6	20.6 ± 2.4	0.925
< 18.5	80 (13.3)	76 (12.4)	90 (15.0)	83 (13.5)	125 (15.9)	0.209
18.5–25	487 (80.9)	505 (82.5)	471 (78.4)	488 (79.1)	624 (79.5)	
> 25	35 (5.8)	31 (5.1)	40 (6.7)	46 (7.4)	36 (4.6)	
Maternal education						<0.0001
No education	359 (60.8)	342 (56.2)	337 (56.3)	353 (57.7)	528 (67.6)	
Informal education	179 (30.3)	205 (33.7)	194 (32.4)	182 (29.7)	167 (21.4)	
≥1 year of formal education	52 (8.8)	61 (10.0)	67 (11.2)	77 (12.6)	86 (11.0)	
Marital status/rank						0.005
Single, divorced, widowed	11 (1.9)	19 (3.1)	13 (2.2)	15 (2.4)	18 (2.3)	
Sole wife	333 (56.4)	294 (48.5)	333 (55.9)	363 (59.3)	390 (49.9)	
1^st^ wife in polygamous household	91 (15.4)	96 (15.8)	99 (16.6)	83 (13.6)	149 (19.1)	
≥2^nd^ wife in polygamous household	155 (26.3)	197 (32.5)	151 (25.3)	151 (24.7)	224 (28.7)	
Adjusted HFIAS^5^	2.96 (2.62, 3.30)	2.61 (2.32, 2.91)	2.98 (2.64, 3.33)	2.62 (2.33, 2.92)	3.11 (2.81, 3.42)	0.354
Asset index	0.10 (0.01, 0.18)	0.15 (0.06, 0.24)	0.04 (-0.04, 0.12)	0.16 (0.07, 0.24)	-0.34 (-0.42, -0.27)	0.288

HFIAS, Household food insecurity access scale; LAZ, length-for-age z-score; positive RDT, positive result of a HRP-II (histidine-rich protein II) rapid diagnostic test for malaria parasites; WAZ, weight-for-age z-score; WLZ, weight-for-length z-score; ZPP, zinc protoporphyrin

^1^P-values are for Chi square tests from survey procedure for comparing proportions in more than 2 categorical variables across groups or logistic regression for categorical variables, and linear mixed model for continuous variables. All comparison were done adjusting for cluster randomization (village, concession);

^2^Mean ± SD, geometric mean (95% CI) and n (%), all such values.

^3^ZPP adjusted categorically for RDT result;

^4^Malaria is defined as a positive result of a HRP-II rapid diagnostic test for malaria parasites

^5^Household food insecurity access scale (HFIAS) adjusted for season.

Caregivers reported high daily adherence to SQ-LNS (96.8% ± 6.5%) and to tablets (97.4% ± 6.4%). Disappearance rates of both products also indicated high weekly adherence [29]. In contrast, during the 12-hr home observations only 62.6% and 54.1% of children at 11 and 16-months, respectively, were observed receiving SQ-LNS during the 12-hr observation periods and 32.0% and 27.1%, respectively, were observed receiving tablets.

Mean baseline Hb was 89 ± 15 g/L, and 91.1% of the children were anemic at enrollment. At 18 months of age, mean Hb concentration increased significantly in IC compared to NIC, with no difference among the four intervention groups (Table 3). This increase in Hb concentration resulted in a final anemia prevalence of 79.1% in IC, compared with 91.1% of children in NIC (P<0.0001). Mean baseline pZn was 69.0 ± 1.2 μg/dL and 35.2% of children had low pZn concentration at enrollment. There was no difference in final mean pZn among any of the study groups nor between IC and NIC. The intervention also did not have a significant impact on mean AGP and CRP concentrations at 18 months, nor on the prevalence of elevated AGP. However, significantly fewer IC children had elevated CRP at 18 months than NIC children (28.9% vs. 41.9%; P = 0.010).

**Table 3:** Hemoglobin, plasma zinc concentration and indicators of inflammation in children at 9 and 18 months^1^.

	LNS-Zn0	LNS-Zn5	LNS-Zn10	LNS-TabZn5	P-value among 4 intervention groups	IC	NIC	P-value between cohorts
**Hemoglobin** ^**2**^								
N at 9 mo	602	613	603	617		2435	785	
Hb at 9 mo (g/L)	89 ± 15	90 ± 16	89 ± 15	90 ± 15	0.735	89 ± 15	88 ± 16	0.417
N at 18 mo	489	498	489	481		1960	664	
Hb at 18 mo (g/L)	97 ± 15	97 ± 15	98 ± 15	98 ± 15	0.613	97 ± 15^a^	88 ± 16^b^	<0.0001
Anemic (Hb <110 g/L) at 18 mo (%)	392 (80.2)	390 (78.3)	384 (78.5)	383 (79.6)	0.848	1549 (79.1)^b^	605 (91.1)^a^	<0.0001
**Adjusted plasma zinc concentration** ^3^ ^,^ ^4^								
N at 9 and 18 mo	84	74	79	73		310	93	
pZn at 9 mo (μg/dL)	68.1 ± 1.21	68.1 ± 1.18	71.0 ± 1.16	71.3 ± 1.21	0.212	69.6 ± 1.19	67.0 ± 1.22	0.090
pZn at 18 mo (μg/dL)	63.5 ± 1.21	64.6 ± 1.18	64.9 ± 1.19	64.8 ± 1.15	0.742	64.4 ± 1.18	64.7 ± 1.18	0.831
Low zinc status (pZn < 65 μg/dL) at 18 mo (%)	50 (59.5)	42 (56.8)	43 (54.4)	34 (46.6)	0.723	169 (54.5)	51 (54.8)	0.425
**C-reactive protein** ^3^								
N at 9 and 18 mo	84	75	79	73		311	93	
CRP at 9 mo (mg/L)	1.94 ± 6.49	3.42 ± 5.35	2.59 ± 5.86	2.97 ± 6.34	0.225	2.65 ± 6.04	2.40 ± 5.87	0.605
CRP at 18 mo (mg/L)	2.64 ± 5.31	2.85 ± 4.83	2.20 ± 4.73	2.12 ± 4.65	0.580	2.44 ± 4.88	3.12 ± 5.50	0.175
Elevated CRP (CRP ≥5 mg/L) at 18 mo (%)	23 (27.4)	27 (36.0)	23 (29.1)	17 (23.3)	0.442	90 (28.9)^b^	39 (41.9)^a^	0.010
**α-1-acid glycoprotein** ^3^								
N at 9 and 18 mo	84	75	79	73		311	93	
AGP at 9 mo (g/L)	1.08 ± 1.27	1.09 ± 1.27	1.08 ± 1.25	1.05 ± 1.33	0.863	1.08 ± 1.28	1.05 ± 1.28	0.325
AGP at 18 mo (g/L)	1.08 ± 1.39	1.07 ± 1.45	1.07 ± 1.41	1.03 ± 1.35	0.880	1.06 ± 1.40	1.11 ± 1.45	0.308
Elevated AGP (AGP ≥1.0 g/L) at 18 mo (%)	49 (58.3)	45 (60.0)	48 (60.8)	41 (56.2)	0.967	183 (58.8)	55 (59.1)	0.829

AGP, α-1-acid glycoprotein; CRP, C-reactive protein; Hb, hemoglobin; IC, intervention cohort; mo, months; NIC, non-intervention cohort; pZn, plasma zinc concentration

^1^ Adjusted mean ± SD, and n (%); all such values. Values in the same row with different superscript letters are significantly different (P<0.05).

^2^ Mean adjusted for cluster randomization (village, concession) and baseline value.

^3^ Venous blood samples collected in a randomly selected sub-group only. Mean adjusted for cluster randomization (village) and baseline value.

^4^ pZn also adjusted for time of blood draw, time since last breastfeeding episode and categorically adjusted for CRP and AGP concentration

**Fig 2:**
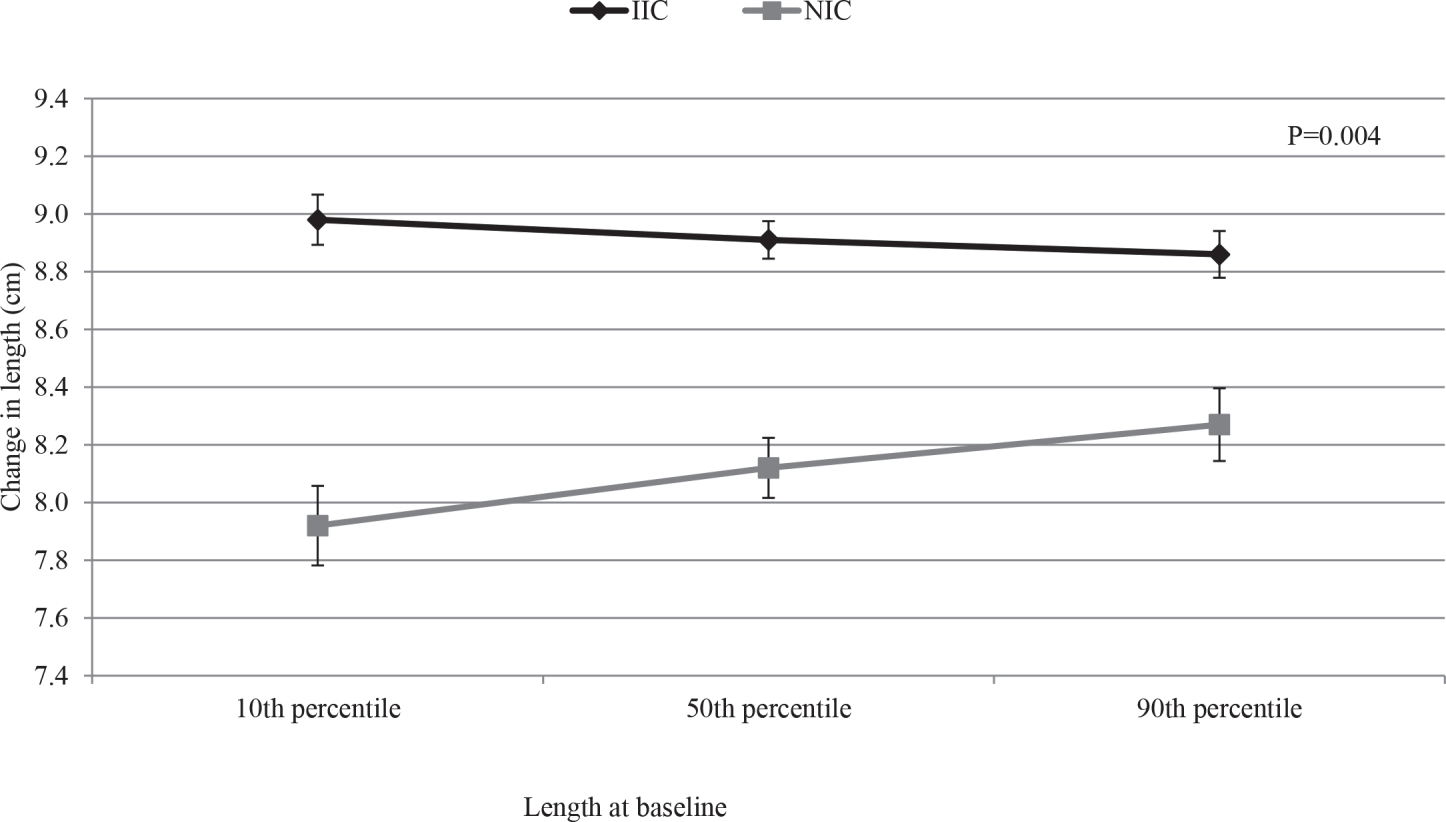
Change in length by initial length in young children in Burkina Faso.

At 18 months, adjusted mean length, weight, MUAC and HC were all significantly greater in IC children compared to NIC children (Table 4), but there were no differences among the 4 intervention groups in any of these outcomes. There was a significant modifying effect of initial length on the difference in growth between IC and NIC children (P = 0.023; **Fig. 2**). In particular, the change in length was significantly greater among IC children with the lowest 10^th^ percentile of initial length compared to NIC children (P<0.0001). At baseline, mean LAZ, WAZ and WLZ were -1.21 ± 1.10, -1.42 ± 1.14, and -0.99 ± 1.05, respectively, in all five groups combined. These z-scores were significantly greater in IC children compared to NIC at 18 months, with no difference among the 4 intervention groups. The increase in length and weight in IC from 9 to 18 months resulted in a significantly lower prevalence of stunting, underweight and wasting in IC compared to NIC. At 18 months, 39.3% of NIC children were stunted compared to 29.3% of IC children (P<0.0001) and 13.5% of NIC children were wasted compared to 8.7% of IC children (P = 0.0003). The prevalence of stunting did not differ among the four intervention groups. However, the prevalence of underweight was marginally higher in children in the LNS-Zn10 group compared to children in the LNS-TabZn5 group (26.3% vs 15.2%, P = 0.089). Similarly, the prevalence of wasting in LNS-Zn10 (13.2%) was significantly higher than in LNS-TabZn5 (5.4%) and LNS-Zn0 (7.2%; P = 0.003). In the 5-group comparison of wasting prevalence, there was no difference between LNS-Zn10, LNS-Zn5 and NIC (P>0.05).

**Table 4:** Anthropometry at baseline and after 9 mo of intervention in young Burkinabe children.

	LNS-Zn0	LNS-Zn5	LNS-Zn10	LNS-TabZn5	P-value among 4 groups^2^	IC	NIC	P-value between cohorts^2^
N	602	613	603	617		2435	785	
Length at 9 mo (cm)^1^	68.7 ± 2.6	68.7 ± 2.6	68.5 ± 2.6	68.9 ± 2.7	0.138	68.7 ± 2.6	68.9 ±2.7	0.393
Length at 18 mo (cm)	77.7 ± 3.0	77.7 ± 3.0	77.7 ± 3.1	77.8 ± 2.9	0.962	77.7 ± 3.0^a^	77.0 ± 3.4^b^	< 0.0001
Weight at 9 mo (kg)	7.41 ± 0.95^ab^	7.41 ± 1.03^ab^	7.30 ± 0.95^b^	7.49 ± 1.03^a^	0.013	7.40 ± 0.99	7.44 ± 1.04	0.625
Weight at 18 mo (kg)	9.31 ± 1.00	9.30 ± 1.09	9.29 ± 1.11	9.33 ± 1.05	0. 784	9.31 ± 1.07 ^a^	9.02 ± 1.18^b^	< 0.0001
MUAC at 9 mo (cm)	13.3 ± 1.1^ab^	13.3 ± 1.2^ab^	13.2 ±1.1^b^	13.4 ± 1.2^a^	0.002	13.3 ± 1.1	13.3 ± 1.2	0.684
MUAC at 18 mo (cm)	13.8 ± 1.0	13.8 ± 1.0	13.8 ± 1.1	13.8 ± 0.99	0.553	13.8 ± 1.0^a^	13.5 ± 1.1^b^	< 0.0001
HC at 9 mo (cm)	42.6 ± 1.4	42.6 ± 1.4	42.6 ± 1.3	42.7 ± 1.3	0.789	42.6 ± 1.4	42.6 ± 1.3	0.330
HC at 18 mo (cm)	44.6 ± 1.4	44.6 ± 1.4	44.6 ± 1.3	44.6 ± 1.3	0.983	44.6 ± 1.3^a^	44.4 ± 1.3^b^	< 0.0001
LAZ at 9 mo	-1.22 ± 1.07^ab^	-1.21 ± 1.11^ab^	-1.33 ± 1.07^b^	-1.14 ± 1.10^a^	0.029	-1.23 ± 1.09	-1.19 ± 1.13	0.631
LAZ at 18 mo	-1.44 ± 1.05	-1.45 ± 1.09	-1.45 ± 1.10	-1.43 ± 1.00	0.968	-1.44 ± 1.06^a^	-1.74 ± 1.17^b^	< 0.0001
WAZ at 9 mo	-1.42 ± 1.10^ab^	-1.41 ± 1.16^ab^	-1.58 ± 1.08^b^	-1.34 ± 1.16^a^	0.003	-1.44 ± 1.13	-1.42 ± 1.19	0.834
WAZ at 18 mo	-1.22 ± 0.94	-1.24 ± 1.00	-1.24 ± 1.04	-1.20 ± 0.94	0.721	-1.23 ± 0.98^a^	-1.51 ± 1.12^b^	< 0.0001
WLZ at 9 mo	-0.99 ± 1.06^ab^	-0.98 ± 1.08^ab^	-1.11 ± 0.99^b^	-0.91 ± 1.06^a^	0.015	-1.00 ± 1.05	-1.01 ± 1.06	0.801
WLZ at 18 mo	-0.72 ± 0.89	-0.74 ± 0.94	-0.77 ± 1.00	-0.70 ± 0.89	0.472	-0.73 ± 0.93^a^	-0.93 ± 1.01^b^	< 0.0001
Stunting at 9 mo, n (%)	139 (23.1)	135 (22.1)	158 (26.2)	124 (20.2)	0.138	556 (22.9)	164 (20.9)	0.318
Stunting at 18 mo, n (%)	146 (29.9)	149 (30.1)	161 (32.8)	117 (24.4)	0.536	573 (29.3) ^b^	261 (39.3)^a^	<0.0001
Underweight at 9 mo, n (%)	159 (26.5)^b^	180 (29.4)^ab^	205 (34.0)^a^	163 (26.4)^ab^	0.015	707 (29.0)	244 (31.1)	0.560
Underweight at 18 mo, n (%)	91 (18.6)	102 (20.4)	129 (26.3)	73 (15.2)	0.051	395 (20.1)^b^	206 (30.9)^a^	0.0001
Wasting at 9 mo, n (%)	100 (16.6)	94 (15.4)	111 (18.4)	88 (14.3)	0.261	393 (16.2)	136 (17.3)	0.674
Wasting at 18 mo, n (%)	35 (7.2)^b^	44 (8.9)^ab^	65 (13.2)^a^	26 (5.4)^b^	0.003	170 (8.7) ^b^	90 (13.5)^a^	0.0003

HC, head circumference; LAZ, length-for-age z-score; mo, months; MUAC, mid-upper arm circumference; WAZ, weight-for-age z-score; WLZ, weight-for-length z-score

^1^ Adjusted mean ± SD and n (%), all such values. Values in the same row with different superscript letters are significantly different (P<0.05).

^2^ Adjusting for cluster randomization (village, concession), baseline value, age and potential co-variates, when applicable.

During the 9 months intervention period in the IC, the mean (95% confidence interval (CI)) prevalence of diarrhea in all four groups combined was 3.08% (2.94, 3.22) and the mean (± SD) diarrhea incidence was 1.10 ± 1.03 per 100 child-days. These did not differ among the four intervention groups (Table 5). The intra-cluster (i.e., concession) correlation for diarrhea incidence was ~0.16, resulting in a design effect of about 1.10 for diarrhea incidence. The proportion of all diarrhea episodes treated according to the study protocol ranged from 36.4% to 41.3% and did not differ by study group. At enrollment, the proportion of children with a positive RDT (61.4% overall) did not differ among groups. During the 9 months follow up, the overall mean malaria prevalence and mean malaria incidence did not differ by group. Almost all malaria episodes were treated (99.0%), and treatment rates did not differ by study group.

**Table 5:** Prevalence and incidence of diarrhea and malaria among young Burkinabe children receiving SQ-LNS and tablets from 9 to 18 months of age.

	LNS-Zn0	LNS-Zn5	LNS-Zn10	LNS-TabZn5	P-value^6^
N	579	593	593	599	
**Prevalence (%)** ^1^					
Diarrhea^2^	3.20 (2.93, 3.47)	3.14 (2.84, 3.44)	2.97 (2.70, 3.24)	3.01 (2.73, 3.29)	0.403
Malaria^3^	1.56 (1.44, 1.69)	1.67 (1.51, 1.83)	1.59 (1.45, 1.73)	1.61 (1.47, 1.76)	0.754
**Incidence (per 100 child-days)** ^1^					
Diarrhea	1.11 ± 1.05	1.14 ± 1.05	1.06 ± 0.98	1.08 ± 1.02	0.589
Treated diarrhea	0.41 ± 0.48	0.41 ± 0.54	0.41 ± 0.51	0.38 ± 0.52	0.711
% of diarrhea episodes treated^4^	39.7	36.4	41.3	37.6	0.330
Malaria	0.55 ± 0.49	0.55 ± 0.52	0.54 ± 0.48	0.52 ± 0.49	0.877
Treated malaria	0.55 ± 0.49	0.54 ± 0.52	0.53 ± 0.48	0.52 ± 0.48	0.813
% of malaria episodes treated^5^	99.2	99.1	99.4	98.3	0.518

^1^Prevalence shown as mean percent (95% CI) and incidence as mean ± SD per 100 child-days. Means are weighted for number of days of observation for prevalence and number of days at risk for incidence.

^2^Diarrhea defined as ≥3 liquid or semi-liquid stools reported by caregiver

^3^Malaria was defined by a positive result of a HRP-II (histidine-rich protein II) rapid diagnostic test for malaria parasites

^4^Treatment of diarrhea included treatments provided by project field workers and health centers

^5^ Treatment of malaria was considered if provided by project field workers

^6^ P-values obtained from binominal regression models, which included a random effect of concession, baseline characteristics and potential covariates and accounted for overdispersion.

There were no significant differences in hospitalization (P = 0.394) or mortality (P = 0.125) rates among children in the four intervention groups. At 18 months, caregivers were asked whether they sought medical services for the study children, and retrospective reports were marginally higher in IC versus NIC (57% vs 54%, P = 0.083), but did not differ among all five study groups (P = 0.412). The mortality rate was significantly lower in IC than NIC (1.4% vs 3.2% P = 0.005). When all five study groups were compared, this difference was only significant between NIC and LNS-Zn5 (**Fig. 1**).

## Discussion

Provision of SQ-LNS along with simple feeding advice and treatment of confirmed cases of malaria and reported diarrhea resulted in significantly greater growth velocity and lower prevalence of stunting, wasting and anemia among children in the IC compared to children in the NIC. The growth effect was significantly greater among children whose initial length was below the 10^th^ percentile of initial length of study participants, indicating that those with the greatest degree of initial growth restriction benefitted most. This reduction in stunting prevalence is of public health significance considering that stunting is associated with increased risk of morbidity and mortality, impaired cognition and educational performance, lower adult wages, and, when accompanied by excessive weight gain later in childhood, increased risk of nutrition-related chronic diseases [1,2].

Within the IC, there were no detectable differences in zinc-related outcomes, such as increased growth, morbidity reduction or change in pZn, regardless of the amount or form of supplemental zinc, implying that the different amounts of zinc provided in SQ-LNS or as dispersible tablet were neither protective nor harmful. Possible explanations for these findings are poor adherence to supplements, poor absorption of zinc from these supplements, deficiencies of other nutrients causing limited responses, or lack of zinc deficiency in the study population. Although reported adherence was very high, 12-hr home observations during home visits revealed that only 59% of children were offered SQ-LNS, and only 30% of the children received a tablet during the 12-hr home observation. Moreover, almost half of all children (46%) who were offered a tablet on the day of observation received the tablet less than 30 min after a meal [29]. Thus, it is possible that adherence to the zinc tablets and/or absorption of zinc from the tablets were less than optimal. It is less likely that concurrent deficiencies of other nutrients limited responses since SQ-LNS provided all IC children with 21 micronutrients and essential fatty acids [11]. Absence of zinc deficiency in the study population is also an unlikely explanation since 54% of children had low pZn at the end of the study.

Although there were no significant differences in mean WLZ among the four intervention groups at the end of the study, the wasting prevalence at 18 months was significantly higher in LNS-Zn10 (13.2%) compared to LNS-Zn0 (7.2%) and LNS-TabZn5 (5.4%; P = 0.003). It is unlikely that this higher wasting prevalence in the LNS-Zn10 group was due to an adverse effect of zinc. A meta-analysis of 22 studies of preventive zinc supplementation provided either as tablets or syrup, including 13 studies that provided 10 mg supplemental zinc, found a small, marginally significant, positive effect of zinc on change in weight-for-height z-score [6]. A previous study found no differences in LNS acceptability or LNS consumption in relation to zinc content [26], so differences in LNS consumption is also an unlikely explanation. Rather, it seems that the difference in final wasting prevalence is a chance finding given that we found no other significant differences in any of the growth and morbidity outcomes among the intervention groups.

While larger quantity LNS has proven useful in the treatment of children with severe acute malnutrition [38], the potential of SQ-LNS to prevent malnutrition and improve health and nutrition status is only beginning to be explored. In the present study, the standardized mean difference of LAZ at the end of the study was 0.27 between the children in IC who received the intervention package compared to children in NIC. Similarly, Adu-Afarwuah and colleagues found a standardized mean difference of final LAZ of 0.26 between Ghanaian children who received SQ-LNS compared to children in a non-intervention group, although the difference in final LAZ at 12 months of age was not significant, probably due to the smaller sample size in the Ghana study [14]. Over the 9-months intervention period we found an effect size in length of 0.48. In contrast, a smaller but also significant effect size of 0.11 was found for the longitudinal average monthly growth effect in Haiti [15]. Possible explanations for these differences are the community-based provision of disease treatment and/or differences in initial LAZ of these study populations. Whereas treatment of diarrhea and malaria was provided by the field team in the present study, no treatment was provided in the studies in Ghana and Haiti. In addition, the mean baseline LAZ was much lower in the Burkinabe children (-1.21 ± 1.10) compared to the baseline LAZ of -0.20 ± 1.0 and -0.39 ± 1.20 in the studies in Ghana and Haiti, respectively. A fourth trial in young Malawian children with mean initial LAZ of -1.00 ± 0.77 found that infants with baseline LAZ below the median who received 50 g LNS gained more length than children who received a micronutrient-fortified maize-soy flour for 12 months [13]. This is consistent with our findings that children with the greatest degree of growth restriction benefitted most.

Diarrhea is associated with a small decrease in linear growth over the long term [39], but findings from longitudinal studies investigating the impact of malaria on growth are inconsistent [40–43]. One study found that only Plasmodium vivax was a predictor of stunting and wasting and not Plasmodium falciparum [44]; the latter was the most common parasite type in our study. Because all children in the intervention groups received SQ-LNS along with simple feeding advice and illness treatment, it is uncertain what proportion of the growth impact in IC children can be attributed to SQ-LNS alone and how much was due to the malaria and/or diarrhea treatment. In the present study, the frequency of treated malaria episodes did not have an impact on average change in growth [45], and it is unlikely that the provision of ORS would have an impact on growth. Nevertheless, to explore the possible effects of illness treatment we compared the results of the present study with the outcomes of another zinc trial in a neighboring health district. In the neighboring study, zinc supplementation was provided only as dispersible zinc tablets along with community-based treatment of malaria and diarrhea, following the same treatment protocol. In that study, where no SQ-LNS was provided, zinc supplements and illness treatment did not increase the growth of young children compared to a non-intervention group [46], suggesting that SQ-LNS may have been the critical growth-promoting component of the current intervention package. However, other differences in the study designs prevent firm conclusions.

We found a significant reduction in anemia prevalence in the IC children. These findings are consistent with the study of SQ-LNS in Ghana, which also found a significant reduction in anemia prevalence in children receiving SQ-LNS from 6 to 12 months of age compared to a non-intervention group [19]. Similarly, studies of MNP also consistently find a beneficial impact on anemia prevalence [47]. However, 79% of all children in IC still had a Hb concentration <110 g/L at the end of the intervention, despite the daily provision of 6 mg iron, 400 μg retinol and B-vitamins in SQ-LNS, along with treatment for malaria and diarrhea. Thus, additional interventions would be needed to reduce the high anemia burden in this population.

Strengths of the current study include the large sample size, high participation rates, and regular training and rigorous supervision of field staff. All anthropometrists participated in regular standardization sessions as recommended by WHO [48]. In addition, the present study included multiple strategies to assess adherence to the study products [29] and blood collection and plasma analyses followed procedures recommended by IZiNCG [31].

Several limitations in the present study also have to be considered. One limitation was the cluster assignment to intervention and non-intervention communities. To compensate for the cluster design, we enrolled a large number of communities (25 IC and 9 NIC villages) and included a design effect of 1.5 in calculating the NIC sample size. Also, adherence to the different study products could not be definitely ascertained despite the use of a variety of strategies to assess adherence [29]. Another weakness of our study is related to the partially blind study design. While participants, field workers and investigators remained blinded to the intervention groups in IC during data collection, data analyses and result interpretation, the comparisons between IC and NIC could not be blinded. However, all final anthropometric indicators were consistently greater in IC and the stunting prevalence of 22.4% at 9 months in all children and 39.3% at 18 months of age in NIC are comparable to the national stunting prevalence in the same age range (25.1% in 9–11 months old children and 41.6% in children aged 18–23 months) in 2010 [49]. Moreover, a similar study in a neighboring health district with weekly contact for morbidity surveillance and product distribution did not find a difference in growth between the intervention and non-intervention group [46]. Thus, it is unlikely that the Hawthorne effect could explain the observed growth impact. Despite these considerations, the inclusion of the NIC in our trial strengthened the study design, as it allowed the comparison of the intervention package to the current standard care in rural southwestern Burkina Faso.

Because the trial was implemented in a malaria-endemic area, SQ-LNS and feeding recommendations for the iLiNS studies were designed to minimize possible risks of iron supplementation [11]. In particular, the daily iron dose was half the recommended dose for MNP [50], and caregivers were advised to feed the daily ration in two separate servings to avoid consuming a large bolus of iron at a single meal. Moreover, treatment was provided for diagnosed cases of malaria and diarrhea. A recent trial of MNP conducted in Pakistan found an increase in reported diarrhea and chest in-drawing among 6 to 18 month old children who received MNP compared to a non-supplemented control group, although there was no difference in the rates of persistent diarrhea, fever, or hospitalizations [51]. Because we did not collect continuous morbidity data in the NIC, so as not to interfere with current treatment practices, the present trial does not allow such a direct comparison of morbidity rates between IC and NIC. In an effort to assess potential risks of the intervention, we asked caregivers at the end of the study to report retrospectively any medical care seeking at local health centers and regional hospitals over the 9-months study period. These reports were marginally higher in the IC (57%) compared to the NIC (54%; P = 0.083), which was likely due to the fact that children in IC were referred to the health center by the morbidity surveillance worker for any illness other than uncomplicated diarrhea and malaria. At the same time, there was a significantly lower mortality rate in IC than NIC (1.4% vs 3.2%; P = 0.005). While our study was not powered to assess hospitalization and mortality rates, these findings together imply that providing SQ-LNS as prescribed in the current study, along with malaria and diarrhea treatment, was likely not harmful.

In summary, there were no differences in growth and morbidity outcomes among the four intervention groups. Thus, the optimal level of zinc to be added to SQ-LNS remains uncertain. Nevertheless, the provision of SQ-LNS along with brief feeding advice and illness treatment of diagnosed cases of malaria and diarrhea had a significant beneficial impact on stunting, wasting and anemia reduction. While we are not able to distinguish the impact of SQ-LNS from the impact of health services, provision of malaria and diarrhea treatment is unlikely the sole reason for the beneficial impact on growth. The findings of the present study suggest that provision of SQ-LNS is a promising strategy to reduce childhood stunting and wasting when coupled with greater access to health care, especially among children at greatest risk of stunting.

## Supporting Information

S1 CONSORT Checklist CONSORT Checklist. (PDF)

S1 Protocol Trial Protocol. (PDF)
