# Supplementary material for: Small-Quantity Lipid-Based Nutrient Supplements, Regardless of Their Zinc Content, Increase Growth and Reduce the Prevalence of Stunting and Wasting in Young Burkinabe Children: A Cluster-Randomized Trial
Source: PLoS One. 2015 Mar 27;10(3):e0122242. doi: 10.1371/journal.pone.0122242 (PMC4376671; doi:10.1371/journal.pone.0122242)
Supplement: S1 Protocol — (PDF) [file pone.0122242.s002.pdf]

Protocol

iLiNS-Zinc

A randomized, double-blind, controlled trial in rural Burkina Faso to determine the optimal amount of zinc to include in a lipid-based nutrient supplement (LNS)

Investigators

Kenneth H. Brown, MD [1,2]

Jean-Bosco Ouedraogo, MD, PhD [3]

Sonja Y. Hess, PhD [1]

et al.

Participating Academic Institutions

[1] University of California, Davis, California, USA

[2] Helen Keller International, Dakar, Senegal

[3] Institut de Recherche en Sciences de la Santé, Bobo-Dioulasso, Burkina Faso

Contact: Prof. Kenneth H. Brown, MD, Program in International and Community Nutrition,
Department of Nutrition, University of California, Davis, CA, USA
Tel. +221 33 869 10 63 , E-mail: khbrown@ucdavis.edu

STUDY PROTOCOL

Principal Investigator: Prof. Kenneth H. Brown, MD

Title of the Study: iLiNS-Zinc. A randomized, double-blind, controlled trial in rural Burkina Faso to determine the optimal amount of zinc to include in a lipid-based nutrient supplement (LNS)

PURPOSE AND PROCEDURES:

This is a multi-center community-based, partially double-blind, placebo-controlled randomized intervention trial. The trial will be a collaborative effort between the Institute of Research in Health Sciences, Burkina Faso (Institut de Recherche en Sciences de la Santé – IRSS,B) and the University of California, Davis, both of which are part of the International Lipid-based Nutrient Supplement (iLiNS) study group, which involves researchers from Burkina Faso, Malawi, Ghana, Finland, France, and the United States. The study is investigator initiated. Funding is provided by a grant from the Bill and Melinda Gates Foundation.

The study uses a cluster sampling design, which consists of 34 communities in the Dandé Health District. Communities will be randomly assigned to either intervention or delayed intervention groups (25 communities in the intervention group, 9 in the delayed intervention group). Within the intervention communities, participants will then be randomized at the level of concession (i.e., the extended family compound) to one of 4 intervention groups as described in more detail below. The total sample size is 3250 children.

Specific aim: The specific aim of the study is to assess zinc-related biochemical and functional responses among young Burkinabe children with a presumed high risk of zinc deficiency who receive micronutrient products (lipid-based nutrient supplements (LNS) or zinc supplements) containing different amounts of zinc, provided with or between meals.

The study has 3 **main objectives**:

1) To determine the lowest daily dose of zinc in LNS that will promote linear growth among 9- to 18-month-old infants in rural communities in Burkina Faso with high rates of stunting.
2) To determine the impact of different zinc doses in LNS on morbidity, micronutrient status, and neuro-behavioral development.
3) To examine the extent to which household food insecurity and other individual, household, and village-level characteristics modify the effects of LNS on child outcomes.

The specific **hypotheses** for the trial are as follows:

1) Young Burkinabe children at risk of zinc deficiency who receive either 5 or 10 mg zinc per day in LNS or 5 mg zinc per day in supplements provided between meals (and LNS to which no zinc has been added) will have greater weight and length increments, decreased incidence of diarrhea and malaria, and a greater increment in plasma zinc concentration and fat-free mass compared with similar children who receive LNS that does not contain added zinc and a placebo tablet.
2) Children who receive LNS (with or without added zinc) or zinc supplements will have greater physical growth (weight and/or length) compared with children in a delayed intervention group.

RESEARCH METHODS

The project will employ the following research methods:

1. Demographic, socio-economic, food security and child feeding practices information will be obtained and analyzed using structured interviews and census data. In a sub-sample, information on child feeding practices will be collected through direct observation.
2. Morbidity data from common infections will be assessed using an interviewer-administered questionnaire, field worker observations, and specific diagnostic tests in the case of malaria.
3. Physical growth will be assessed by measurement and analysis of change in anthropometric dimensions.
4. Body composition will be assessed through measurement and analysis of percent body fat and fat-free mass, using a stable isotope (deuterium) dilution technique.
5. Biochemical indicators of nutritional status will be assessed by collection and analysis of blood, saliva and urine samples.
6. Neurobehavioral development will be evaluated by assessment of developmental milestones and through comprehensive tests of development.
7. Dietary pattern will be assessed using food frequency questionnaires.
8. Adherence to the supplement regimens will be assessed by questionnaires and fieldworker observation.

Demographic, socio-economic, food security and child feeding practices

An initial census will be conducted to map and enumerate households in the study area. Indicators of socio-economic status (occupation, housing and possessions) will be assessed during the census in all households through survey questionnaires administered by field workers. Eligible participants will be identified through the census, which will be repeated periodically to identify newborns. Information on household and village characteristics and household food security will be collected through survey questionnaires administered by field workers upon enrollment and every six months thereafter to capture seasonal variability in participating households. In a 10% sub-sample, field workers will visit participants in their homes to obtain information on care- and feeding practices and child activity patterns through observation of usual household activities during two 12-hour home visits.

Morbidity surveillance

In the intervention communities, field surveillance workers will visit each home in their assigned villages on a weekly basis during the intervention period to assess morbidity from common infections. Diarrhea will be defined as the presence of four or more loose or liquid stools per 24 hours, as reported to field workers by caregivers. Temperature and two one-minute respiratory rates will be measured by field workers on the first week of the month and whenever fever is reported or respiratory distress is observed. Upper respiratory infection will be defined as the presence of cough and purulent nasal discharge, and lower respiratory infection by the presence of cough and age-specific increase in respiratory rate (>50/minute for infants <12 months of age and >40/minute for older children). New episodes of diarrhea and respiratory infections will be considered when at least 72 hours have elapsed since a previous episode has resolved. Malaria will be assessed by the Rapid Diagnosis Test (HRP2, histidine-rich protein 2) and both thick and thin blood smears.

Anthropometry

The weight, length, right mid-upper arm circumference, and head circumference of all participants will be measured at baseline in children in both the intervention and non-intervention communities. The weight and length information will allow calculation of the prevalence of stunting (low height-for-age), underweight (low weight-for-age), and wasting (low weight-for-height). Mid-upper arm circumference is a useful parameter for analyzing nutritional status in children aged 6-59 months because it measures muscle mass and can be used as a proxy for wasting. Head size, in part, measures brain growth. Maternal height and weight will also be measured at baseline in both the intervention and non-intervention communities; we will adjust for maternal anthropometric measures in analyses of child growth.

Biological sample (blood) collection and laboratory analysis

Micronutrient status of the study population will be assessed by measurement of biochemical markers in blood. Hemoglobin and zinc protoporphyrin concentration will be measured in a drop of capillary blood in all children. Zinc, retinol, retinol-binding protein, plasma ferritin, transferrin receptor, C-reactive protein, and alpha-1 acid glycoprotein concentrations will be measured in plasma processed from venous blood samples in a randomly chosen sub-sample of children in each study group (total sample size of biochemical sub-group: n=626). In the same sub-sample, a few drops of whole blood will be stored on filter paper to assess thyroid hormone status and thyroglobulin concentration.

Anemia will be assessed by measuring hemoglobin in all children. A drop of capillary blood will be obtained by finger stick from each child for hemoglobin assessment using a portable Hemocue photometer (Hemocue, Inc; Lake Forest, CA). Children with hemoglobin <50 g/L will be excluded from the study and referred for further evaluation and treatment. Children with moderate anemia (hemoglobin 50 – 79 g/L) will be treated according the national policy. Namely, they will receive iron supplementation (3-6 mg iron/kg/d for at least 30 days) and an anthelmintic treatment (Mebendazole). These latter participants will remain in the study during and after the additional iron and anthelmintic treatment, and will continue to receive LNS and zinc/placebo supplements, but will be specifically identified in the analyses.

Zinc status of children will be assessed by measuring plasma zinc concentrations by atomic absorption spectrometry (Shimadzu AA-6300; Tokyo, Japan). Vitamin A status of children will be assessed by measuring retinol-binding protein and by measuring plasma retinol determined by reverse-phase high performance liquid chromatography. Iron deficiency will be assessed by measuring erythrocyte zinc protoporphyrin in unwashed and washed red blood cells using a hematofluorometer (Aviv Biomedical, Lakewood, NJ, USA) and by measuring plasma ferritin and transferrin receptor. Iodine and thyroid status indicators (thyroxin, thyroid-stimulating hormone and thyroglobulin concentrations) will be assessed in dried blood spots on filter paper by two-site dissociation enhanced lanthanide fluorescent immunoassay (Delfia) (Perkin Elmer Life Sciences, Wallac, Turku, Finland).

C-reactive protein and alpha-1 acid glycoprotein are acute phase proteins which are elevated during periods of infection. Because concentrations of nutritional status indicators may decrease (zinc and retinol) as a result of infection, the presence of an acute phase reaction can confound the interpretation of these nutritional status indicators. Therefore, these two proteins will be assessed at the time of each blood sampling to control for the presence of an acute phase reaction and to make it possible to interpret the values for minerals and vitamin A status indicators. Plasma ferritin, transferrin receptor, retinol-binding protein, C-reactive protein and alpha-1 acid glycoprotein concentrations will be measured by ELISA (Erhardt et al, Journal Nutrition, 2004). Appropriate quality assurance techniques will be implemented in the laboratory at IRSS, and a sub-set of samples will be reanalyzed in the reference laboratory at the University of Otago or UC Davis.

When the child is ill or has had diarrhea or fever during any of the past two days of a scheduled baseline collection of blood, urine and saliva, the child will not be included in the biochemistry subgroup. Final collection of blood, urine, and saliva will be postponed if the child has a fever >38.5 °C or is acutely ill. In the case of the final biochemical sample, supplementation will be continued until the sample is obtained.

Biological sample (urine) collection and laboratory analysis

In a subset of children (n=626), body composition will be assessed using deuterium dilution to measure total body water. Body composition assessment permits determination of the proportions of total body weight that are comprised of fat mass, lean mass (bone, muscle), and water.

On the day of these studies, infants will be allowed to breastfeed upon arrival to the health center, and a baseline urine and saliva sample will be obtained. An oral dose of deuterium oxide of 4 g will be administered, and saliva samples will be obtained post dosing. All food and beverages consumed between the time of dosing and the final saliva sample will be recorded. The child will be weighed before

and after breastfeeding episodes to estimate breast milk consumption. Percent body fat will be calculated to determine the percentage of total body weight that is made up of fat.

Iodine status will be measured in the same subgroup of children (n=626). The urine sample will be stored for later assessment of urinary iodine concentration using a modification of the Sandell-Kolthoff reaction.

Neurobehavioral development

Motor, social, and language development will be assessed every 4 weeks by asking the mother selected questions from the Denver Development Screening Test 2. The following questions (timing of the acquisition of these skills) have previously been validated and shown to differentiate 6-18 month old infants in other studies in Africa: unsupported sitting, unsupported standing, supported walking, unsupported walking, running, pronouncing single words like mama / dada, waving goodbye, eating by self, drinking from a cup. The chosen motor development questions (except running) are also promoted by the World Health Organization (WHO). In addition to collecting data on maternal report of the above mentioned motor development milestones, their achievement will be confirmed by observation every 3 months. More detailed information on neuro- behavioral development will be based on comprehensive tests of development performed at 18 mo of age in a randomly selected subgroup of children (600 children from the delayed intervention group and 1350 children from the intervention group [450 children for each of the groups LNS-Zn0, LNS-Zn10, and Suppl-Zn5]).

Motor, language, and personal-social development at age 18 months is being evaluated using a adapted version of the Developmental Milestones Checklist (DMC) by Abubakar et al. (Acta Pediatr 2010;99:291-97). Since we have modified the published version of the checklist, we will evaluate the developmental sensitivity of the adapted tool. In addition to the regularly scheduled 18 month assessment, we will administer the checklist to a total of 200 children in the intervention group at a second time point: 50 children at age 12 months, 50 at age 15 months, 50 at age 21 months, and 50 at age 24 months. These data will provide a cross-sectional estimate of the increase in DMC scores from age 12 to 24 months. If any effect of LNS supplementation is found on DMC scores, we will use this estimate to translate the effect size into the developmental advance in units of months of age.

Dietary patterns

Food frequency questionnaires covering the period of the previous 24 hours and the previous 7 days will be assessed in all children at 9 and 18 months. For all children in the intervention groups, a shorter version of the food frequency questionnaire will also be administered monthly to assess continuation of breastfeeding and every 3 months to determine the frequency of consumption of zinc-containing foods.

Adherence

Each week during the home visits the surveillance worker also will assess adherence to the study protocol by measuring any leftover LNS and zinc/placebo supplements. This will be done prior to providing the caregiver with a new one-week supply of LNS and zinc/placebo supplements for the child. In addition, the use of LNS and zinc/placebo supplements will be observed during 12-hour in-house observations. In a 10%-subsample, observers will spend 12 hours in the home and record all supplement related behaviors, including who received the products and ways in which the LNS was given.

Procedures:

Sensitization

Once all approvals are obtained, a series of meetings will be convened with local political authorities at the regional, district and sub-district levels, and with village leaders in each of the study villages. The purpose of these meetings between study staff and community leaders are to explain the objectives of the study and to obtain their permission to continue with recruitment in those communities. Caretakers of

young children who show a preliminary interest in the trial will be invited to an information session for further description of the trial design and requirements and a preliminary screening for enrollment criteria.

Census

After obtaining community support to conduct the study, an initial census will be conducted to map and enumerate households in the study area. Eligible participants will be identified through periodic censuses in the study area.

Acceptability testing

Before implementing the proposed study, the acceptability of the LNS products will be confirmed by field testing. Once the acceptability by infant and mother of the LNS products has been proven in the sensory evaluations, the products will be prepared by Nutriset (a food company based in Malauney, France) and delivered to IRSS,B in coded packages labeled specifically for this study.

Recruitment and enrollment

Individual communities will be stratified by selected indicators (size; proximity to road and the city of Bobo-Dioulasso; and health clinic affiliation) and then randomly assigned within strata to have children participate in the intervention from 9-18 months (~75% of communities) or from 18-27 months (~25% of communities).

Nine-month-old healthy infants will be identified by the periodic censuses in the study area. Children aged 9 months of age whose caretakers show a preliminary interest in the trial will be invited to an information session for further description of the trial design and requirements.

Written, informed consent will be obtained from all study families by study supervisors, using culturally appropriate explanations of all study procedures, benefits, and risks. Separate consent forms will be used for participants in intervention communities and the delayed intervention communities to better describe the respective interventions. An additional consent form will be administered in the biochemical sub-group to obtain written consent for any future analyses in the collected biochemical samples.

Children whose parents sign the consent will undergo a preliminary screening (described below) to determine if they meet the enrollment criteria. Those children meeting all enrollment criteria will be given a trial identification number. Children in the communities that participate in the intervention will be randomly assigned at the level of the concession (i.e., the extended family compound) to one of the four intervention groups.

The age of the participants will be confirmed from eligible subjects' road-to-health under-five cards, which provide the date of birth. Identity cards of each participant will be produced to facilitate identification of the participants.

The inclusion criteria are:

- Signed informed consent from at least one caretaker
- Age 9.0 months to 9.9 months
- Permanent resident in Dandé Health District, Burkina Faso
- Planned availability during the period of the study
- Acceptance of home visitors

The exclusion criteria are:

- Hemoglobin <50 grams/liter
- Weight-for-height <70th percentile of the NCHS reference growth charts
- Presence of bipedal edema
- Severe illness warranting hospital referral

- Congenital abnormalities potentially interfering with growth
- Chronic medical condition (e.g. malignancy) requiring frequent medical attention
- Infant with known HIV infection or infant from mother with HIV infection
- History of allergy towards peanuts
- History of anaphylaxis or serious allergic reaction to any substance, requiring medical care
- Concurrent participation in any other clinical trial
- Absence from the field site for more than 3 weeks

Data collection

The data collection team will consist of one post-doctoral fellow, one laboratory coordinator, and two medical officers supervising a team of 6 field supervisors, 25 field workers, 4 anthropometry teams, two phlebotomy teams, and six data entry clerks.

Data will be collected in three phases: 1) screening and baseline assessment (at 9 months of age), 2) surveillance during intervention (9-18 months of age), 3) and post-intervention assessment (at 18-42 months of age).

Screening and baseline assessment

All eligible children 9 months of age whose caregivers consent to participate will be examined by a medical doctor or nurse. Children will be excluded from participation in the study and referred to the health clinic for routine health services if the health history and physical exam reveal the presence of the following conditions: severe anemia (Hb <50.0 g/L); severe wasting (weight-for-height <70th percentile of the NCHS reference growth charts; bipedal edema; severe illness warranting hospital referral; chronic medical conditions including HIV infection (or if the child's mother is infected with HIV); history of peanut allergy (because the LNS contains peanuts); or history of anaphylaxis or serious allergic reaction to any substance, requiring emergency medical care.

In the intervention groups, anthropometric measurements (as detailed previously in the Methods section) will be taken on all children upon enrollment. Biochemical assessments (blood, urine, and saliva) will be conducted at enrollment in a sub-sample (n=626). An initial morbidity screening will be conducted at the clinic following the information session.

The screening at 9 and 18 months of age will be identical for intervention and non-intervention communities. However, the children in the delayed intervention group will not be visited during the 9-month supplementation period. Thus, the study team will not be available to provide any treatment services or referrals, and the families will use the same medical services that are ordinarily available. The local services consist of a system of health centers and district-level referral hospitals, which offer services according the WHO program for the Integrated Management of Childhood Illnesses (IMCI). IMCI is a simple algorithm based on clinical symptoms and signs that allows relatively untrained village health workers to diagnose and provide treatment for common childhood illnesses (diarrhea, acute lower respiratory tract infection, malaria, acute malnutrition) and referral for complicated cases. Currently, treatment of uncomplicated diarrhea in the community consists of oral rehydration solution and dietary counseling for continued feeding. Children with dehydration, dysentery, or persistent diarrhea are referred to the nearest health center for more extensive evaluation and treatment, including intravenous therapy and antibiotics when indicated. The reason for including the delayed intervention communities is to help with the interpretation of the growth data in the intervention communities. Children in the delayed intervention group will receive LNS with 10 mg zinc from the age 18 to 27 months, as described in Table 1.

In an effort to keep the anthropometrists, phlebotomists and other fieldworkers unbiased in their data collection activities, the study staff will be informed that the children in the delayed intervention communities are measured for census purposes only and that they are not part of the intervention study.

Interventions

After completion of baseline assessments, the children in the intervention groups will begin receiving the respective masked, color-coded intervention products (LNS and placebo or zinc supplements) daily for 9 months. The supplements will be distributed weekly by a field worker who will also assess the disappearance rate of the LNS and the supplements by estimating how much remains (0%, 25%, 50%, 75%, 100%) in the children's homes at the end of each week. Empty packages and any unconsumed supplements from the previous week will be gathered as markers of adherence.

Approximately, 2440 infants from the intervention communities who meet set criteria will be randomly assigned to receive the following intervention from 9 to 18 months of age:

1) LNS without zinc, placebo supplement (LNS-Zn0): 0 mg zinc in 20 grams LNS per day and a daily placebo supplement between meals from age 9 to 18 months.
2) LNS with 5 mg zinc, placebo supplement (LNS-Zn5): 5 mg zinc in 20 grams LNS per day and a daily placebo supplement between meals from age 9 to 18 months.
3) LNS with 10 mg zinc, placebo supplement (LNS-Zn10): 10 mg zinc in 20 grams LNS per day and a daily placebo supplement between meals from age 9 to 18 months.
4) LNS without zinc, 5 mg zinc supplement (Suppl-Zn5): 0 mg zinc in 20 grams LNS per day and a 5 mg zinc supplement between meals from age 9 to 18 months.

In addition, approximately 810 infants from the delayed intervention communities who meet set criteria will be assigned to the following group:

5) Standard treatment with delayed LNS intervention (ST-DI): Standard care from age 9 to 18 months; LNS containing 10 mg zinc per 20 grams LNS, delayed until age 18 to 27 months.

This information is summarized below in Table 1.

Table 1. Intervention groups and regimens

Study group and child age	**Contents of intervention products**	
	**LNS**	**Zinc or placebo supplement**
**Age 9-17 mo.**		
LNS-Zn0	0 mg zinc in 20 g LNS/day	Placebo daily
5	5 mg zinc in 20 g LNS/day	Placebo daily
n10	10 mg zinc in 20 g LNS/day	Placebo daily
Suppl-Zn5	0 mg zinc in 20 g LNS/day	5 mg zinc supplement daily
ST-DI	Standard care	Standard care
**Age 18-27 mo.**		
LNS-Zn0	Standard care	Standard care
LNS-Zn5	Standard care	Standard care
LNS-Zn10	Standard care	Standard care
Suppl-Zn5	Standard care	Standard care
ST-DI	10 mg zinc in 20 g LNS/day	No supplement
**Age 28-42 mo.**		
LNS-Zn0	Standard care	Standard care
LNS-Zn5	Standard care	Standard care
LNS-Zn10	Standard care	Standard care
Suppl-Zn5	Standard care	Standard care
ST-DI	Standard care	Standard care

The LNS will be delivered in plastic pots containing 140 g (sufficient for one week) or sachets containing 20 g (the dose for one day) and labeled individually with one of 8 color codes (two colors per treatment group) to avoid the possibility of any mix-ups during delivery to the household. The child's caregivers will be provided with a measuring spoon and advised to feed the day's allotment (20 g = 2 spoonfuls) in two servings at meal times (1 spoonful per each of two meals). In particular, the caregiver will be requested to add 1 spoonful of LNS to ~2 tablespoons of cereal porridge or other complementary food and then to continue feeding any desired additional amount of the food. This is to maximize the likelihood that all of the prescribed LNS dose will be served each meal.

All mothers will be encouraged to continue breastfeeding on demand.

The LNS will be produced and packed by Nutriset (Malaunay, France). Raw ingredients include peanut paste, dried skimmed milk, maltodextrin, sugar, vegetable oil, and a mineral and vitamin mix. The products are packed in opaque, plastic containers, each containing 140 g of the supplement or foil sachets containing 20 g of the LNS. LNS containers will be stored in the Bama field office at a temperature between 20˚C and 40˚C. At these temperatures, the nutritional quality is guaranteed for 12 months. Because of low water activity, the products are resistant to microbial contamination. The specific nutrient content of each is summarized in Table 2.

Table 2: Serving size and nutrient composition of the food supplements for the trial

Nutrient	LNS-Zn0	LNS-Zn5	LNS-Zn10
Ration (g/day)	20	20	20
Total energy (kcal)	124	124	124
Protein (g)	2.6	2.6	2.6
Fat (g)	9.6	9.6	9.6
Linoleic acid (g)	4.5	4.5	4.5
α-Linolenic acid (g)	0.58	0.58	0.58
Vitamin A (µg RE)	400	400	400
Vitamin C (mg)	30	30	30
Vitamin B_1(mg)	0.3	0.3	0.3
Vitamin B_2 (mg)	0.4	0.4	0.4
Niacin (mg)	4	4	4
Folic acid (µg)	80	80	80
Pantothenic acid (mg)	1.8	1.8	1.8
Vitamin B_6 (mg)	0.3	0.3	0.3
Vitamin B_{12} (µg)	0.5	0.5	0.5
Vitamin D (IU)	200	200	200
Vitamin E (mg)	6	6	6
Vitamin K (µg)	30	30	30
Iron (mg)[1]	6	6	6
Zinc (mg)	0.3	5.3	10.3
Cu (mg)	0.2	0.2	0.2
Calcium (mg)	280	280	280
Phosphorus (mg)	190	190	190
Potassium (mg)	200	200	200
Magnesium (mg)	40	40	40
Selenium (µg)	20	20	20
Iodine (µg)	90	90	90
Manganese (mg)	1.2	1.2	1.2
Phytate (mg)	n/a*	n/a*	n/a*

* n/a: Information not available.

[1]Target iron content has been reduced from 9 mg in Nutributter to 6 mg, to reduce any potential risk associated with iron intake in malarial areas. As the daily ration will be divided into at least 2 meals during the day, the amount of iron consumed at any single meal should not exceed 3 mg. This falls within the amount of iron that a fortified processed complementary food would provide at a given meal, which is considered acceptable by the World Health Organization/ United Nations Children's Fund (WHO, 2007)).

Zinc supplements will be provided as water-dispersible tablets produced by Nutriset (Malaunay, France) containing 5 mg of zinc per day or an identical product without zinc. The caregivers will be advised to provide the supplements once daily, either between meals or with breast milk, but not with other foods. The supplements will also be labeled individually with one of 8 color codes (the same colors per treatment group as for LNS) to avoid the possibility of any mix-ups during delivery to the household. Samples of the LNS and zinc supplements/placebos will be culled systematically for analysis of zinc content in an independent laboratory.

LNS supplementation will continue for 9 months, starting after baseline screening at nine (9.0 – 9.9) months and ending after 9 months when the participants are 18 (18.0 – 18.9) months old. For the delayed intervention group, LNS containing 10 mg of zinc per 20 g will be given from 18 to 27 months of age.

<u>Other treatments provided to study participants</u>

Recommendations on infant feeding
All groups, including the delayed intervention group, will receive simple messages on infant feeding from the study personnel at the time of baseline and final screening (age 9 and 18 months). The messages will include continued breast feeding, and in the intervention communities, instructions on how to feed LNS and the supplements.

Health care
The study participants will be advised to attend health centers according to the same routine schedule as all other Burkinabe infants and receive the usual services provided through the national health system. Participants with documented peripheral blood malaria parasitemia at any point of the trial will be treated with nationally recommended antimalarial drugs, currently a combination of artesunate and amodiaquine. Children with diarrhea will be treated with oral rehydration solution (ORS) packets and advised to continue feeding. Children with signs of dehydration, dysentery, or persistent diarrhea (>14 d) will be referred to the health center. The children presenting with symptoms of malaria or diarrhea will not be removed from the study. They will continue to receive the daily LNS and zinc/placebo supplement, in addition to whatever treatment is provided by their physician.

Participants with documented severe anemia (hemoglobin < 50 g/L) at any point of the trial will be referred for blood transfusion at national health facilities and will be excluded from the study at baseline. Participants with moderate anemia (hemoglobin between 50 and 79 g/L) will be treated according the national policy. Namely, they will receive iron supplementation (3-6 mg iron/kg/d for at least 30 days) and an anthelmintic treatment (Mebendazole). Participants requiring treatment at any timepoint during the study will remain in the study during and after the additional iron and anthelmintic treatment and will continue to receive LNS and zinc/placebo supplements, but will be specifically identified in the analyses. All other medical conditions will be treated by the Burkina Faso's health system, according to the standard national guidelines.

<u>Surveillance during intervention and post-intervention data collection</u>

a. <u>Intervention groups</u>

   In the intervention groups, the families will receive the respective supplements at weekly intervals and the participants will undergo regular surveillance as described above. The scheduled data collection is summarized below in Table 3.

b. <u>Delayed-intervention group</u>

Children in the delayed intervention communities will be screened at baseline and at 18 months of age only (Table 4). During the 9-month supplementation period, the children in the delayed intervention group will not be visited.

Table 3. Summary of data collection in relation to time of enrollment into the study - Intervention groups (LNS-Zn0, LNS-Zn5, LNS-Zn10, Suppl-Zn5)

Child age (months)	09	10	11	12	13	14	15	16	17	18	19	20	21	22	23	24	25	26	27	28	30	36	42
Food supplement delivery (weekly)	x	x	x	x	x	x	x	x	x	x													
Morbidity and adverse events (weekly)	x	x	x	x	x	x	x	x	x	x													
Anthropometry	x			x			x			x													
Hemoglobin	x									x													
ZPP (capillary)	x																						
Malaria (capillary and venous for biochemical subgroup)	x									x													
Biochemistry (venous and capillary ZPP, zinc, retinol, retinol-binding protein, plasma ferritin, transferrin receptor, CRP, AGP; thryroid hormones, thyroglobulin in subgroup)	x									x													
Body composition (saliva in subgroup)	x									x													
Urinary iodine (subgroup)	x									x													
Socio-economic status	x						x						x						x				x
Full food frequency questionnaire	x									x													
Household food security	x						x																
12-hour observation care & feeding practices (subgroup)		x						x															
Developmental milestones (reported) and breastfeeding questions	x	x	x	x	x	x	x	x	x	x													
Developmental milestones (observed) and zinc food questions	x			x			x			x													
Full developmental assessment (subgroup)										x													
Caregiver knowledge, attitudes, practices (KAP)							x																

Table 4. Summary of data collection in relation to time of enrollment into the study - Delayed intervention group (ST-DI)

Child age (months)	09	10	11	12	13	14	15	16	17	18	19	20	21	22	23	24	25	26	27	28	30	36	42
Food supplement delivery (monthly)										x	x	x	x	x	x	x	x	x	x	x			
Morbidity	x																						
Anthropometry	x									x													
Hemoglobin	x									x													
ZPP (capillary)	x																						
Malaria (capillary and venous in biochemical subgroup)	x																						
Biochemistry (venous and capillary ZPP, zinc, retinol, retinol-binding protein, plasma ferritin, transferrin receptor, CRP, AGP; thryroid hormones, thyroglobulin; subgroup)	x									x													
Body composition (saliva and urine in subgroup)	x									x													
Urinary iodine (subgroup)	x									x													
Socio-economic status	x									x			x						x				x
Food frequency questionnaire	x									x													
Household food security	x																						
12-hour observation care & feeding practices (subgroup)																							

Developmental milestones (reported) and breastfeeding questions																							
Developmental milestones (observed) and zinc food questions	x									x													
Full developmental assessment (subgroup)										x													
Caregiver knowledge, attitudes, practices (KAP)							x																

Data entry and analysis

All data collection forms will be reviewed by the field supervisors for accuracy and completeness and then transferred to the data entry clerks for double data entry in the field office. The database program will incorporate range and consistency checks. Data will be transferred weekly to IRSS,B (the main coordination office of the study) and will be backed up by internet upload (via a secure connection) to a protected folder on the UC Davis Smartsite. Programs will be developed to detect any outlier data. All data collected by each field worker also will be checked for mean values and number preference, and any retraining will be scheduled, if necessary. The field workers will meet with the field supervisors weekly to discuss any problems that may arise.

Analysis will be on an intention-to-treat basis. Thus, once enrolled, all subjects will be included in the analysis until the time they complete the study, drop out voluntarily, or are withdrawn, for pre-defined reasons. Withdrawn or discontinuing participants are not replaced by others; i.e. possible drop-outs do not influence the sample size, once the study has started. If caregivers refuse blood drawing, other data will still be included in the analysis.

Statistical analysis will be completed with SAS for Windows. The distribution of each outcome variable will be examined, and variables will be transformed as needed to meet the assumptions of normality of residuals and homogeneity of variance. If no suitable transformation can be found, non-parametric techniques will be used. Baseline characteristics of intervention groups will be compared to determine whether any variables differ among groups despite randomization. In addition, baseline characteristics of subjects who are lost to follow-up will be compared with those of subjects who complete the study to assess potential bias due to attrition.

For the primary outcomes of change in length-for-age Z-score, incidence of diarrhea and malaria and change in plasma zinc concentration between 9 and 18 months, the analysis will compare the difference (95% confidence intervals) between means of the control and intervention groups (LNS-Zn5, LNS-Zn10, Suppl-Zn5 versus LNS-Zn0 and all intervention groups versus the delayed intervention control group) using analysis of covariance, controlling for any baseline variables that either differ between groups or are predictive of the outcome.

A test for interaction and stratified analyses will be done to explore whether the effects of zinc on the primary or secondary outcomes are modified by certain baseline characteristics. Variables in this analysis will include the participant's initial length, weight, sex, plasma zinc concentration, erythrocyte zinc protoporphyrin concentration, maternal education, number of siblings, as well as the household food security and income indicators.

Proportion of child deaths before age 18 months and proportion of child drop-out before age 18 months will be compared across the four intervention groups by Fisher's exact test.

Frequency of analyses

The main statistical analysis will be done after the last participant attains the age of 18 months and the main intervention is completed. First the dataset will be checked for outliers. Then participants will be assigned to their respective treatment group to allow group-wise analysis. The randomization code revealing the identity of the assigned group will be broken only after the statistical analysis of the main outcomes is completed and only after consulting among all investigators. A further analysis will be done at the end of the follow-up, i.e., when all children reach 42 months of age.

Nature, frequency and duration of tests

In all children, capillary blood samples will be obtained by finger stick upon enrollment (9 months of age) and at 18 months of age to measure hemoglobin and malaria parasites count. At 9 months, the finger stick will also be used to assess ZPP and malaria antigen. In the biochemistry subgroup, capillary ZPP will also be done at 18 months.

Biochemical indicators of micronutrient status will be assessed in a randomly selected sub-set of children (n=626). For these subjects, caregivers will bring their children to the clinic for the baseline and final specimen collections. Venous blood samples (5 ml whole blood) will be obtained from subjects at the baseline and final clinic visits. The samples will be drawn in the morning at the local community health center. When the child is ill or has had diarrhea or fever during the past two days of a scheduled baseline collection of blood, urine and saliva, the sample collection will be postponed until

the symptoms resolve. Final collection of blood, urine, and saliva will be postponed if the child has a fever >38.5 °C or is acutely ill. In the case of the final biochemical sample, supplementation will be continued until the sample is obtained.

In the intervention groups, the length, weight, right mid-upper arm circumference and head circumference will be measured for all children at the baseline, 12, 15 and 18 months of age during the intervention. The same anthropometric measurements will be completed for children in the delayed intervention group at baseline and 18 months of age. We anticipate that clinic visits including blood sample collection and anthropometry will take approximately 4-5 hours to complete.

In the "biochemistry" subset of children, body composition will be assessed twice, at baseline and 18 months of age, at the health center. We anticipate that the body composition assessment will take approximately 4-5 hours.

Comprehensive tests of development will be performed once at 18 months of age in a randomly selected subgroup of children (600 children from the delayed intervention group and 1350 children from the intervention group [450 children for each of the groups LNS-Zn0, LNS-Zn10, and Suppl-Zn5]). Motor, social, and language development will be assessed every 4 weeks through selected questions from the Denver Development Screening Test 2 during home visits. We anticipate that the tests of development administered in the homes will take approximately 15 minutes to complete. In addition, at baseline, 12, 15 and 18 months of age during the intervention children's achievements of the motor development milestones will be observed by the anthropometry team. We anticipate that this additional test will take approximately 10-15 minutes.

<u>Location and duration of the study.</u>

The study will be conducted in the Dandé Health District in Houet Province of southwestern Burkina Faso, West Africa. Dandé is located 56 kilometers from Bobo-Dioulasso. This area has been selected both because of high rates of nutritional stunting and because the IRSS,B has developed excellent rapport with local community leaders and residents.

In total, the duration of the study will be 33 months. The data collection will run for about 48 months. Data analysis and dissemination of results will occur over 24 months.

We anticipate that the duration of the study will be 4 years (including census, baseline data collection, intervention, follow-up, laboratory and data analysis). Subjects will be included in the intervention group will be included in the study for a period of approximately 3.5 years but will receive the intervention for only 9 months of that time. Recruitment is anticipated to begin in April 2010, with data collection lasting until the end of June 2012. Laboratory analyses and data entry are expected to be completed by December 2012, and data analysis will occur by June 2013.

<u>Plan to monitor data to ensure subject safety</u>

All data collection forms will be reviewed by the field supervisors for accuracy and completeness and then transferred to the data entry clerks for double data entry in the field office. The database program will incorporate range and consistency checks. Programs will be developed to detect any outlier data.

Individuals who experience a serious adverse event that is likely to be related to the trial intervention, become seriously ill, meet the criteria for severe wasting (weight-for-height <70th percentile of the NCHS reference growth charts), or severely violate the protocol will be withdrawn from the study by the investigator (but will be included in the analysis up to the time of their exclusion).

If known, the reason for withdrawal will be recorded. Children who are withdrawn on the basis of development of wasting will be referred to the nearest nutritional supplementary feeding program and, if necessary, the child and their families assisted with transportation.

Field workers will complete detailed morbidity assessments for children enrolled in the study, facilitating close follow-up of subjects' health status by field workers during the intervention period. Through these ongoing assessments, field workers will monitor children for the presence of fever, diarrhea, vomiting, cough, nasal discharge, respiratory distress, skin rash, depressed appetite, and any other symptoms of concern to the care giver. Temperature and two one-minute respiratory rates will be measured whenever fever is reported or respiratory distress is observed at the time of the home visit and once monthly when these symptoms are absent. In the case of fever (tympanic temperature >38°C) or report of fever

since the previous morning, the presence of malaria will be assessed by the Rapid Diagnosis Test and treated according to the national guidelines as indicated. The field workers will be trained to recognize dehydration and other danger signs, and to refer all children with dehydration, dysentery, fever, or persistent diarrhea to the local health clinic for medical evaluation. Serious adverse events that are determined by the study physicians to be “related” or “likely related” to the study intervention will be reported immediately to the principal investigators, who will notify the chairs of their respective Institutional Review Boards. Serious adverse events that are determined by the study physicians to be “unlikely related” to the study intervention will be reported to the principal investigators on a monthly basis and summarized for the Institutional Review Boards in yearly progress reports.

RESOURCES TO CONDUCT THIS STUDY

The project is fully supported by a grant from the Bill and Melinda Gates Foundation. To implement this program and ensure the ultimate application of the research findings, a consortium, the iLiNS study group, has been formed among researchers from Burkina Faso, Malawi, Ghana, Finland, France, and the United States. These groups have complementary expertise in the technical aspects of zinc nutrition, implementation of community-based nutrition and disease treatment programs, and evaluation research. Specifically, technical experts from the Program in International and Community Nutrition (PICN) of UC Davis, Helen Keller International, and the Institute of Research in Health Sciences (Institut de Recherche en Sciences de la Santé - IRSS) in Bobo Dioulasso, Burkina Faso will collaborate on the design, implementation, and evaluation of this project.

UC Davis will provide administrative, financial and technical oversight. A post-doctoral research fellow in International and Community Nutrition will be present at the field site, serving as field nutrition coordinator throughout the period of data collection; and the Statistician of the PICN will assist with data analysis and the preparation of final statistical models. A sub-contract will be prepared with IRSS, Bobo-Dioulasso, which will be responsible for implementation of the field work, including recruiting and hiring of the field research team, data entry and processing, and laboratory analyses. The core study team will coordinate the day-to-day management of the study out of the field office at the Dandé District Hospital. Technical experts from International Zinc Nutrition Consultative Group (IZiNCG), which is housed administratively within UC Davis and based physically in the HKI regional office in Dakar, Senegal, will assist in ensuring quality control of the zinc analyses, and will assist with dissemination of the results of the study and subsequent advocacy with the Ministry of Health in Burkina Faso. Supervision will be provided through regular field visits and conference calls by the senior investigators and prinicipal investigators of the study team. Professor Brown, who is presently based in Dakar, where he also serves as the HKI Africa Regional Advisor for Nutritional and Child Survival, will be able to make frequent supervisory visits to the field site in Burkina Faso.

Nutriset S.A.S. has developed and will produce the lipid-based nutrient supplements (LNS) and zinc/placebo supplements used in this trial. It will also be responsible for the quality control of the products.

The IRSS,B is equipped with the following facilities and equipment:

- High speed internet connection with WIFI
- Mono and bi-distillation machine
- Millipore Direct-Q: ultra pure water
- Autoclave
- Nutrition/Chemical: HPLC, Atomic absorption spectrophotometer, FTIR
- Serological: ELISA reader, beta counter and luminescence
- Radioimmunoassay: hybridization oven, beta counter
- Parasitology: microscopes
- Insectarium
- Chemical room for plant compound extraction
- Cell culture room with hoods, incubator, centrifuges, cell harvester
- Molecular biology: thermocyclers, electrophoresis, photo documentation system

Research Personnel

UC DAVIS RESEARCH PERSONNEL

Name	Title	Department
Kenneth H Brown	Professor	UC Davis Department of Nutrition
Sonja Y Hess	Associate Research Nutritionist	UC Davis Department of Nutrition
Stephen A Vosti	Associate Adjunct Professor	UC Davis, Dept. of Ag. and Res. Econ.
Souheila Abbeddou	Post-Doctoral Scholar	UC Davis Department of Nutrition
Elizabeth Prado	Post-Doctoral Scholar	UC Davis Department of Nutrition

NON-UC DAVIS RESEARCH PERSONNEL

Name	Title	Department
Jean-Bosco Ouédraogo	Professor	Institut de Recherche en Sciences de la Santé
Jerome Somé	Research Assistant	Institut de Recherche en Sciences de la Santé
Rosemonde Guissou	Research Assistant	Institut de Recherche en Sciences de la Santé
Zinewendé Ouédraogo	Research Assistant	University of Ouagadougou
Elizabeth Yakes	Assistant Professor	University of New Mexico

Role of each key member of the study personnel.

a. The Principal Investigator (KHB) and the Co-Principal Investigator (JBO) will serve as study contact at UCD/HKI and IRSS,B, respectively, throughout project implementation, will assist with study design, analysis, and interpretation of the results, and will support the resolution of administrative issues. The Principal Investigator of health economics (SAV) will assist with study design, analyses and interpretation of the results of the socio-economic and health economics aspects of the study. In addition, the Co-Principal Investigator (JBO) will be responsible for obtaining national, regional, and local consent at district and community level as well as subsequent advocacy with the Ministry of Health in Burkina Faso.

b. The UC Davis technical advisor (SYH) will assist with study design, analysis, supervising field work, interpretation of the results and the dissemination of the results of the study.

c. The UC Davis postdoctoral fellows (SA, EY) will assist with study design, analysis, and interpretation of the results, and be responsible for direct supervision of the field work and data entry and processing at the field site.

d. The medical officers (ZO, JS) will be responsible for obtaining local consent at district and community level. They will assist with analysis and interpretation of the morbidity results and will be responsible for medical referral and follow up in case of illness of a study participant.

e. The postdoctoral fellow (SA), field coordinator (RG), and medical officers (ZO, JS) will be present at the field site throughout the period of data collection, and will be responsible for implementation of the field work, including training of the field research team, supervising data entry and processing, and laboratory analyses.

SUBJECT SELECTION:

The target population for enrollment includes healthy, 9-month-old infants who live in the Dandé Health District, Houet Province, Burkina Faso, and meet all of the inclusion and none of the exclusion critieria.

All potential participants will receive information about the study during their enrollment visit using a group discussion with opportunities for questions and discussions. Those interested in participating will be invited to a private discussion with study personnel, during which the potential participants may ask questions about the study. Those wishing to participate will then sign an informed consent form, indicating the voluntary nature of the study and the participants' right to discontinue follow-up at any point.

3250 children will be enrolled in this study for baseline and final assessment. Approximately 2440 (610 per group) will be enrolled in the intervention groups, and approximately 810 will be enrolled in the delayed intervention group.

The research subjects will include infants aged 9.0 months to 9.9 months upon enrollment. These children will be followed for 33 months, until attaining a maximum 42 months of age. The age of the participants will be confirmed from eligible subjects' road-to-health under-five cards, which provide the date of birth. Where documentation is not available, a timeline of important local events will be used to estimate subjects' birthdates.

<u>The inclusion criteria are:</u>
- Signed informed consent from at least one caretaker
- Age 9.0 months to 9.9 months
- Permanent resident in Dandé Health District, Burkina Faso
- Planned availability during the period of the study
- Acceptance of home visitors

<u>The exclusion criteria are:</u>
- Hemoglobin <50 grams/liter
- Weight-for-height <70th percentile of the NCHS reference growth charts
- Presence of bipedal edema
- Severe illness warranting hospital referral
- Congenital abnormalities potentially interfering with growth
- Chronic medical condition (e.g. malignancy) requiring frequent medical attention
- Infant with known HIV infection or infant from mother with HIV infection
- History of allergy towards peanuts
- History of anaphylaxis or serious allergic reaction to any substance, requiring medical care
- Concurrent participation in any other clinical trial
- Absence from the field site for more than 3 weeks

RISKS:

The risks involved in this study are minimal. Although nutritional status can be assessed by clinical examination, biochemical assessment was selected because it permits assessment of multiple micronutrients over the full range of adequate status to mild, moderate, or severe deficiency.

Blood draws can cause momentary pain and discomfort and can result in bruising, but rarely result in infection or other complaints. Blood draws will be performed by qualified health professionals. Two capillary blood samples will be collected from all subjects at ages 9 and 18 months. In a sub-set of subjects, two 5-ml venous blood samples will be collected when participants are 9 and 18 months old. The collected volume represents <1% of the child's total blood volume and is replaceable from the child's own blood production within a day; the total volume of blood collected (10 ml) is minimal and unlikely to result in any adverse effects.

The study poses risks of psychological harm to participants as a result of inadvertent lack of respect for cultural norms, which is likely to be unpleasant but temporary. The risk will be minimized by consulting with local community leaders prior to the start of the study, and obtaining their permission to recruit subjects in their villages. IRSS,B, established in 1997, has developed excellent rapport with local community leaders and residents. Additionally, the majority of the study team members are from Burkina Faso and have extensive field experience that will be shared during training.

There is a risk of accidental release of personal health information which may have important social or psychological consequences for the subject. This risk will be minimized by the confidentiality measures described below.

<u>Plan for protecting subject privacy and confidentiality.</u>

Subject data will be identified with unique identification numbers, and full names will not be attached to the data. All documentation regarding the participants, including the laboratory samples, source data and the Adverse Event Form will be identified with appropriate participant codes, both on paper and in computer files. The full names will only appear on informed consent forms and a separate coding list.

The investigators shall maintain the regulatory documents (informed consents, ethical approval) until participants reach the age of 25 years. Selected data (participants' name, home village, household identification number, enrollment date, and enrollment number) will be summarized in an electronic database and a paper participant register. The registers will be stored in a locked space at the study office. Data from these registers may be given only to authorized members of the research team and authorities guiding health research in Burkina Faso and at the University of California, Davis.

Subject names will not be included in any data entry or reporting of results. Blood collection will be completed in a private area of the health center to ensure subject privacy. Additionally, interviewers will request that only the respondents be present during the interviews conducted during home visits. Subject privacy and confidentiality practices will be addressed during training of all study personnel.

Plan for reporting adverse and serious adverse events to the IRB

All suspected adverse events are documented according to standard operating procedures devised in conjunction with the Institutional Review Boards at IRSS,B and the University of California, Davis. Suspected serious adverse events that are determined by the study physicians to be "related" or "likely related" to the study intervention are immediately reported to the principal investigators, who will notify the chair of the respective Institutional Review Boards. Serious adverse events that are determined by the study physicians to be "unlikely related" to the study intervention will be reported to the principal investigators on a monthly basis and summarized for the Institutional Review Boards in yearly progress reports.

Data will be collected for suspected adverse events during the intervention period from enrollment to 18 months of age in the intervention groups . Potential adverse reactions to study foods are elicited with structured forms at the weekly home visits. Serious adverse events include all untoward medical occurrences that result in death, are life-threatening, require inpatient hospitalization, result in persistent or significant disability, or are otherwise considered a serious medical condition by a study physician. Information on the causes of death will be collected by a verbal autopsy method.

Use of placebo

This study involves the use of a placebo because of the possibility of "regression to the mean", whereby individuals with lower initial plasma zinc concentrations, morbidity or growth status subsequently have higher ones and *vice-versa*. Therefore, it will be possible to interpret the effects of supplementation only if there is a placebo comparison group.

The purpose of the delayed intervention group is to control for possible functional responses to LNS that does not contain added zinc and/or a "Hawthorne effect" due to the presence of the study team, either of which could affect growth rates independently of LNS and zinc.

BENEFITS*:*

All participants will receive free food supplements during the intervention or delayed intervention periods. Approximately three-fourths of the participants will receive free food supplements (LNS) between 9 and 18 months of age and the rest will receive free food supplements (LNS) between 18 and 27 months of age. All participants will further benefit from assessment of nutritional status and referral for treatment by usual services when necessary.

All participants receive the benefit of learning whether or not they are anemic. Each subject will be given their hemoglobin value immediately after their blood is collected. Staff will explain whether their hemoglobin is in a normal range or low (anemic) range and refer subjects with severe anemia to the health center for treatment.

Participants in the intervention groups will benefit from weekly morbidity surveillance and selected treatment or referral, as indicated, from 9 to 18 months of age. If needed, the research team will give the participants in the intervention groups treatment for specific illnesses or refer them for treatment at the local health center for specific serious health conditions. All participants who are found to have a blood hemoglobin concentration below 50 g/l will be referred for blood transfusion according to national treatment guidelines and excluded from the study. Participants with hemoglobin between 50 and 79 g/l will be treated for anemia (iron syrup and Mebendazole) free of charge.

Aside from receiving LNS and zinc supplementation, assessment of health and nutritional status, and referral for treatment when necessary, there is no other direct benefit to the subject.

RISK-BENEFIT RATIO:

The study involves minimal risk to subjects. The psychological and social risks of participation are unlikely and/or temporary, and the physical risks from blood draws are temporary and not serious. Participants will benefit individually by receiving free food supplements, assessment of health and nutritional status, and being tested for anemia and referred to a treatment center as needed.

The results of the study may offer a partial solution to a major public health problem in Burkina Faso and other Sub-Saharan countries. Should the intervention work, it will significantly contribute to the options for community-based approaches to prevent malnutrition and stunting among young children in the intervention communities. Internationally, the results will be relevant to help develop the optimal formulation of LNS and similar products.

COSTS/COMPENSATION TO SUBJECTS:

The study will not involve any added expenses to the subject or a third party. The study will involve time costs to the participants and their primary caregivers to complete eligibility and consent, in-home interviews, and travel to the health center for blood sample collection and anthropometric assessment. However, the most time-intensive portion of the study, weekly interviews, will occur within the subjects' homes for their convenience. The time is justified in that subjects will receive an anemia diagnosis, overall assessment of health and nutritional status, weekly screening of morbidity, and diarrhea and malaria treatment if indicated. The general population will likely benefit from the information derived from this study.

Participants will be compensated for any travel expenses related to the visit at the health center for anthropometric assessment and blood collection. Each time a child is invited to the health center (baseline, anthropometry and final assessments), the caregiver who accompanies the child will be compensated with 500 FCFA (approximately USD $1).

Any adverse events arising during the study will be treated within the national health system, i.e., there is no special health insurance for the study participants. In case of life-threatening circumstances, the study team will provide assistance in transportation to appropriate national health care facilities.
